# Artificial Photosynthesis: Current Advancements and Future Prospects

**DOI:** 10.3390/biomimetics8030298

**Published:** 2023-07-09

**Authors:** Abniel Machín, María Cotto, José Ducongé, Francisco Márquez

**Affiliations:** 1 Divisionof Natural Sciences and Technology, Universidad Ana G. Méndez-Cupey Campus, San Juan, PR 00926, USA; 2 Nanomaterials Research Group, Department of Natural Sciences and Technology, Universidad Ana G. Méndez-Gurabo Campus, Gurabo, PR 00778, USA; mcotto48@uagm.edu (M.C.); jduconge@uagm.edu (J.D.)

**Keywords:** photoelectrochemical cells, hydrogen evolution reaction, oxygen evolution reaction, CO_2_ fixation

## Abstract

Artificial photosynthesis is a technology with immense potential that aims to emulate the natural photosynthetic process. The process of natural photosynthesis involves the conversion of solar energy into chemical energy, which is stored in organic compounds. Catalysis is an essential aspect of artificial photosynthesis, as it facilitates the reactions that convert solar energy into chemical energy. In this review, we aim to provide an extensive overview of recent developments in the field of artificial photosynthesis by catalysis. We will discuss the various catalyst types used in artificial photosynthesis, including homogeneous catalysts, heterogeneous catalysts, and biocatalysts. Additionally, we will explore the different strategies employed to enhance the efficiency and selectivity of catalytic reactions, such as the utilization of nanomaterials, photoelectrochemical cells, and molecular engineering. Lastly, we will examine the challenges and opportunities of this technology as well as its potential applications in areas such as renewable energy, carbon capture and utilization, and sustainable agriculture. This review aims to provide a comprehensive and critical analysis of state-of-the-art methods in artificial photosynthesis by catalysis, as well as to identify key research directions for future advancements in this field.

## 1. Introduction

Natural photosynthesis is an efficient process in which green plants, algae, and certain bacteria convert light energy, typically from the sun, into chemical energy in the form of glucose [1,2]. This process occurs in chloroplasts, more specifically within a complex called the photosystem, where light absorption triggers a series of electron transfer reactions that, in turn, generate ATP and NADPH. These molecules are then used to fix carbon dioxide into glucose during the Calvin cycle [1,2,3].

Artificial photosynthesis attempts to mimic this natural process to create an efficient, clean, and cost-effective way to convert sunlight into storable energy forms, mainly hydrogen or other solar fuels [2]. Generally, this is performed by developing photo-electrochemical cells that absorb light and split water into hydrogen and oxygen or by using solar energy to drive the reduction of carbon dioxide into carbon-based fuels [3,4,5]. This endeavor, however, faces challenges because the technology to catalyze these reactions at a reasonable cost and with a high level of efficiency is still in development.

Table 1 presents a comparison between natural and artificial photosynthesis. Comparatively, although both natural and artificial photosynthesis harness sunlight, they differ significantly in their implementation and efficiency. Natural photosynthesis is highly optimized through billions of years of evolution, achieving an efficiency of around 3–6% in converting sunlight into stored chemical energy. In contrast, artificial systems currently struggle to achieve similar efficiencies at a comparable cost [6]. However, artificial photosynthesis holds the promise of producing energy-dense fuels, unlike natural photosynthesis, which primarily produces glucose. Thus, though still under development, artificial photosynthesis has the potential to be a sustainable solution for our energy needs, helping to address the challenges of climate change and energy security [7,8,9,10]. 

The main objective of this review is to provide a comprehensive overview of the recent advancements and challenges in the field of artificial photosynthesis (see outline below). We will be discussing the principles, materials, strategies for performance enhancement, integration, and potential future applications that enable the substantial improvement of processes from an eco-sustainable perspective. This review intends to provide a valuable resource for researchers, engineers, and policymakers working in the field of renewable energy and environmental sustainability.

## 2. Photoelectrochemical Cells

**Figure 1:**
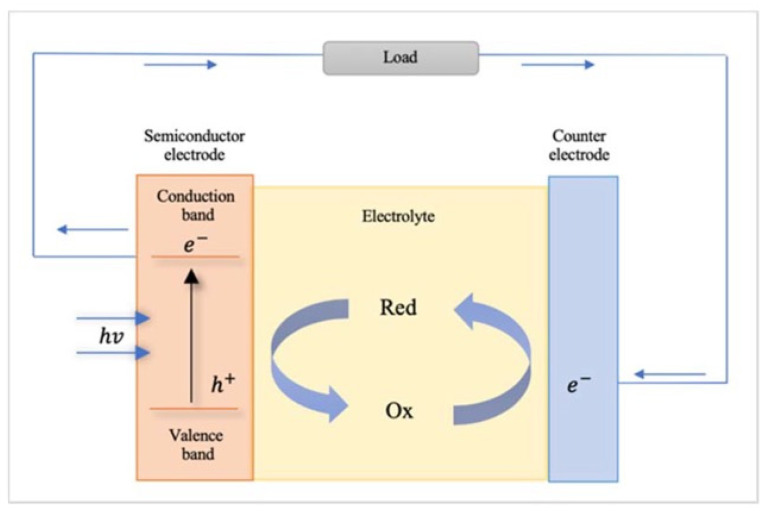
Schematic of a photochemical cell. (Reprinted with permission from Ref. [2], Copyright 2021, Materials-MDPI).

Photochemical cells are essential components of artificial photosynthesis systems, as they directly convert solar energy into chemical energy [1]. These cells consist of a light-absorbing material, catalysts, and redox mediators that facilitate the conversion of absorbed photons into chemical reactions, such as water splitting and carbon dioxide reduction. The development of efficient and stable photochemical cells is crucial for the success of artificial photosynthesis technology and its potential applications [2,3]. Figure 1 presents the schematic of a photochemical cell. 

In photochemical cells, the process of artificial photosynthesis begins with the absorption of light by a photosensitizer, a light-absorbing material that generates excited electrons upon illumination. The photosensitizer can be organic dye, inorganic dye, or a quantum dot, each with their unique light absorption characteristics [4,5]. The efficiency of the photosensitizer is determined by its ability to absorb a broad range of the solar spectrum and its excited state lifetime, which influences the charge separation process. Upon light absorption, the excited electrons are transferred from the photosensitizer to a suitable electron acceptor while the holes (h^+^; positive charges) are transferred to an electron donor. This charge separation process is essential for converting the absorbed light energy into chemical energy and avoiding the rapid recombination of generated charges, which would result in energy loss [6,7,8].

The separated charges drive two critical reactions in artificial photosynthesis: water oxidation and carbon dioxide reduction. In water oxidation, also known as the oxygen-evolving reaction (OER), the holes generated during the charge separation process oxidize water molecules to produce oxygen gas and protons [9,10]. In carbon dioxide reduction, also known as the carbon dioxide reduction reaction (CO_2_RR), excited electrons reduce CO_2_ to produce value-added chemicals and fuels, such as carbon monoxide, formic acid, methanol, or methane. The performance of a photochemical cell in these reactions is determined by the activity, selectivity, and stability of the catalysts used for water oxidation and CO_2_ reduction [11].

The electron transfer process in photochemical cells is facilitated by redox mediators, which shuttle electrons between the photosensitizer and the catalysts. Redox mediators can be metal complexes or organic molecules, and their role is to minimize energy loss during electron transfer and prevent charge recombination. In addition, redox mediators can affect the selectivity of the CO_2_RR by controlling the potential and the number of electrons transferred to the CO_2_ molecule [12,13,14]. The final step in artificial photosynthesis is the formation of the desired products, which can be hydrogen gas, value-added chemicals, or fuels. The product distribution is determined by the thermodynamics and kinetics of the catalytic reactions, as well as the local concentration of reactants and products. In some cases, the photochemical cell is integrated with a membrane separator or a gas-diffusion electrode to facilitate the separation of the products and increase the overall efficiency of the system [15,16].

### 2.1. Materials in Photochemical Cells

#### 2.1.1. Photosensitizers

Organic dyes, such as metalloporphyrins, phthalocyanines, and ruthenium polypyridyl complexes, have been widely used as sensitizers in dye-sensitized solar cells (DSSCs) because of their strong absorption coefficients and high molar extinction coefficients. In a study conducted by Mathew and group [17], a molecularly engineered porphyrin dye, coded SM315, which features the prototypical structure of a donor–π-bridge–acceptor and both maximizes electrolyte compatibility and improves light-harvesting properties, was used in DSSCs. They found that using SM315 with the cobalt (II/III) redox shuttle resulted in dye-sensitized solar cells that exhibited a high open-circuit voltage VOC of 0.91 V, short-circuit current density J_SC_ of 18.1 mA cm^–2^, fill factor of 0.78, and a power conversion efficiency of 13%. Even when organic dyes are relatively inexpensive and offer tunable absorption properties, their long-term stability and limited light-harvesting efficiency remain challenges [17,18]. Recent advances in molecular engineering have resulted in the development of new organic dyes with improved performance and stability [18,19]. 

Inorganic dyes, such as cadmium sulfide (CdS) and cadmium selenide (CdSe), have also been employed as sensitizers [20] for their higher stability and broader absorption spectra compared to organic dyes, but their toxicity and potential environmental impacts remain major concerns [21]. Perovskite materials, which have demonstrated remarkable efficiency improvements in solar cells, can also be considered inorganic dyes and have been used as promising materials [22]. Yoo and group [22] reported using a holistic approach to improve the performance of PSCs through enhanced charge carrier management. First, they developed an electron transport layer with film coverage, thickness, and composition by tuning the chemical bath deposition of tin dioxide (SnO_2_). Second, the authors decoupled the passivation strategy between the bulk and the interface, leading to improved properties, while minimizing the bandgap penalty. The devices exhibited an electroluminescence external quantum efficiency of up to 17.2% and an electroluminescence energy conversion efficiency of up to 21.6%. As solar cells, they achieved a certified power conversion efficiency of 25.2%, corresponding to 80.5% of the thermodynamic limit of its bandgap.

Quantum dots, or semiconductor nanocrystals, have also emerged as promising sensitizers for artificial photosynthesis systems as a result of their unique optical properties, such as a size-tunable bandgap and multiple exciton generation [23,24,25]. They have shown improved efficiencies compared to organic dyes, but their toxicity and potential environmental impacts remain major concerns. Recent studies have focused on developing alternative, less toxic quantum dot materials, such as copper indium sulfide (CIS) and silver indium sulfide (AgInS_2_) [23]. For example, researchers [23] developed CuInS_2_ (CIS)-based solar cell devices by sensitizing TiO_2_ photoanodes with CIS quantum dots (CISQDs). The research group reported a maximum efficiency of 3.8% (with JSC ≈ 6.2 mA, VOC ≈ 926 mV and FF ≈ 66 for cell area ≈ 0.25 cm^2^ and thickness ≈ 20 µm) when 4.6 nm CISQDs that were sensitized on composite photoanode were used. The group explains that the high VOC observed was possible because of the combined effect of the P25 composite photoanode’s properties (such as fewer defects, good connectivity between particles, effective light scattering, and minimum recombination) with an effective electron transport and the size of the optimized CuInS_2_QDs. 

Silicon-based mesoporous materials also play an important role in the design and implementation of photoelectrochemical cells for artificial photosynthesis because of their inherent semiconductor properties, high surface area, and controllable pore size [2,4,5,6]. Silicon, particularly in its nanostructured form, possesses direct band gaps that facilitate efficient charge transfers [2]. Furthermore, silicon’s natural abundance and non-toxicity contribute to the sustainability and potential large-scale applications of these systems [7,8].

Recent advances in mesoporous silicon fabrication technologies, such as electrochemical etching and magnesiothermic reduction, have allowed for the creation of highly ordered, crystalline structures that are beneficial for photon absorption and charge transportation [9,14]. Additionally, strategies for the modification of mesoporous silicon, such as doping with other elements or coupling with suitable co-catalysts, have been explored for improving its photoelectrochemical performance [6]. These efforts have demonstrated promising results in enhancing the stability and efficiency of silicon-based photoelectrochemical cells, paving the way for practical applications of artificial photosynthesis [9].

#### 2.1.2. Catalysts

Another approach that has been employed is the use of molecular catalysts as a result of their ability to facilitate a redox reaction that converts solar energy into chemical energy [26]. Examples of molecular catalysts for artificial photosynthesis include transition metal complexes, such as cobalt (Co) [27], manganese (Mn) [28], and iron-based complexes [29]. These catalysts offer the advantage of cost-effectiveness and sustainability compared to noble metal catalysts, but their catalytic activity and stability often lag behind [27,28,29,30]. Recent research efforts have focused on developing more robust molecular catalysts with improved performance and stability [29,30,31]. A study conducted by Wolff and group [31] reported simultaneous H_2_ and O_2_ evolution by CdS nanorods decorated with nanoparticulate reduction and molecular oxidation co-catalysts. The authors explained that the process proceeded entirely without sacrificial agents and relied on the nanorod morphology of CdS to spatially separate the reduction and oxidation sites. They further explained that hydrogen was generated on Pt nanoparticles grown at the nanorod tips, whereas Ru(tpy)(bpy)Cl_2_-based oxidation catalysts were anchored through dithiocarbamate bonds onto the sides of the nanorod. In the case of O_2_ generation from water, the research group explained that the process was verified using ^18^O isotope-labeling experiments, and time-resolved spectroscopic results confirmed efficient charge separation and ultrafast electron and hole transfer to the reaction sites. The authors ended by arguing that the system demonstrated that combining nanoparticulate and molecular catalysts on anisotropic nanocrystals can provide an effective pathway for visible-light-driven photocatalytic water splitting.

Nanostructured catalysts, such as metal oxides, metal sulfides, and metal-organic frameworks (MOFs), have also been explored for artificial photosynthesis applications. These materials offer a high surface area and tunable electronic properties, making them attractive candidates for catalytic applications [32,33,34,35,36,37]. Examples of nanostructured catalysts for artificial photosynthesis include cobalt oxide (Co_3_O_4_) [35], nickel oxide (NiO) [36], and iron sulfide (FeS_2_) [37]. Alam and group [37] reported Pyrite (FeS_2_)-decorated 1D TiO_2_ nanotubes in a bilayer as a sustainable photoanode for photoelectrochemical water splitting activity. The results of the catalyst (15-FeS_2_@TiO_2_) showed a higher photocurrent density of 1.59 mA/cm^2^ at 0.3 V versus a reference electrode of Ag/AgCl (or at 1.23 V versus a reversible hydrogen electrode) using a 100 mW/cm^2^ intensive light source and a donor density (N_D_) of 3.68 × 10^−13^ cm^−3^ as compared to that of pure TiO_2_NTs (0.09 mA/cm^2^), 05-FeS_2_@TiO_2_NTs (0.19 mA/cm^2^), 10-FeS_2_@TiO_2_NTs (0.53 mA/cm^2^), and 20-FeS_2_@TiO_2_NTs (0.61 mA/cm^2^). The authors explained that the photoelectrochemical activity results were attributed to the homogenous integration of FeS_2_ that not only increased the charge separation but also intensively interacted with the substrate (TiO_2_ nanotubes), which resulted in excellent photoelectrochemical activity. Even when these materials offer the advantages of cost-effectiveness and sustainability, their catalytic activity and stability are often less than those of noble metal catalysts [32]. 

#### 2.1.3. Electron Mediators

Cobalt-based redox mediators, such as cobalt bipyridine and cobalt phenanthroline complexes, have been widely used in artificial photosynthesis systems as a result of their favorable redox properties and stability [38,39]. These mediators can efficiently shuttle electrons between the photoanode and the counter electrode, reducing the overall overpotential of the system and improving its efficiency. However, cobalt-based mediators can suffer from high recombination rates and limited diffusion coefficients, which can adversely impact their overall performance [40]. Copper-based redox mediators, such as copper phenanthroline and copper bipyridine complexes, have also been explored as alternatives to cobalt-based mediators in artificial photosynthesis systems [41]. Copper-based mediators offer several advantages, such as a lower cost and abundant availability compared to cobalt mediators. They also demonstrate good electron transfer properties and stability. However, their catalytic activity and stability may not be as high as those of cobalt-based mediators, and their applications in artificial photosynthesis systems require further optimization [42,43]. Organic redox mediators, such as organic molecules containing viologen, TEMPO, and ferrocene moieties, have also been investigated for use in artificial photosynthesis systems [44]. These mediators offer several advantages, including a low cost, good solubility, and tunable redox properties, but their long-term stability and compatibility with other materials in the system remain challenges [45,46]. Recent research efforts have focused on developing new organic mediators with improved stability and performance for artificial photosynthesis applications [47].

### 2.2. Strategies for Enhancing Photochemical Cell Performance

#### 2.2.1. Strategies for Enhancing Photochemical Cell Performance in Artificial Photosynthesis

**Figure 2:**
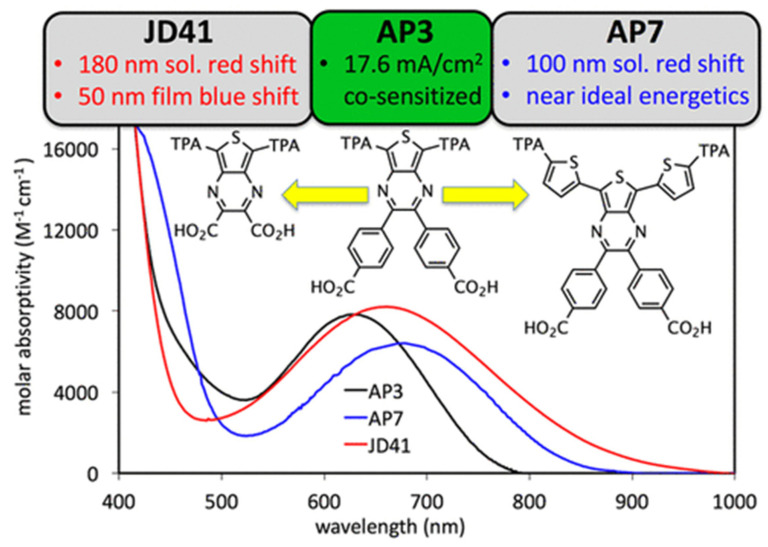
Use of near−infrared−absorbing organic dyes in dye-sensitized solar cells. (Reprinted with permission from Ref. [52], Copyright 2017, Journal of Organic Chemistry-American Chemical Society).

One approach to enhance the performance of photochemical cells is broadening their absorption spectra, which allows them to capture more sunlight and convert it into useful energy. This can be achieved by designing novel photosensitizers with extended absorption profiles, employing multiple photosensitizers with complementary absorption spectra, or introducing additional light-harvesting materials into the system [48,49,50,51]. For example, Cheema et al. [52] synthesized and characterized seven organic sensitizers, employing thienopyrazine (TPz) as a π-bridge in a double donor, double acceptor organic dye design (see Figure 2). The author reported that the thienopyrazine (TPz) building block allows for NIR photon absorption in dye-sensitized solar cells (DSCs) when used as a π-bridge and that the dye design was found to be remarkably tunable with solution absorption onsets ranging from 750 to nearly 1000 nm. Furthermore, the incorporation of quantum dots with tunable absorption properties has been shown to improve the light-harvesting capabilities of photochemical cells [53].

Plasmonic enhancement is another strategy for improving the light-harvesting efficiency of photochemical cells. Plasmonic nanoparticles, such as gold and silver, can concentrate and scatter light, leading to enhanced absorption by photosensitizers [54]. Several studies have demonstrated the benefits of incorporating plasmonic nanoparticles into photochemical cells, resulting in increased power conversion efficiencies [55,56]. Liu and group [57] reported on the integration of gold nanoparticles (Au NPs) into the mesoporous TiO_2_ layer of dye-sensitized solar cells, obtaining a power conversion efficiency of 6.4%, which was significantly higher than a TiO_2_ DSSC. The short circuit current density was increased by 23% and the conversion efficiency was improved by 28% with the addition of Au NPs. This improvement is attributed to the increase in light harvesting efficiency and lower charge carrier recombination rate of the TiO_2_-Au DSSC.

**Figure 3:**
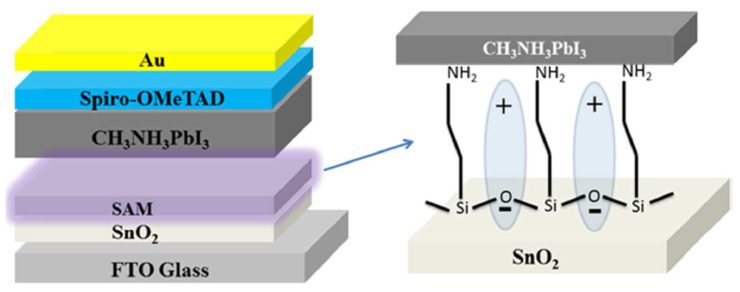
Schematic diagram of a perovskite solar cell based on the SAM-modified SnO_2_ ESL. (Reprinted with permission from Ref. [61], Copyright 2017, Journal of Materials Chemistry A-Royal Society of Chemistry).

The performance of photochemical cells can also be improved by optimizing the interfaces between various materials in the system. Proper interface engineering can enhance charge separation and transport rates, reduce recombination losses, and, ultimately, increase overall efficiency [58]. This can be achieved by introducing additional layers, such as holes or electron transport layers, or by modifying the interface with functional groups or molecules [59,60]. For instance, Yang et al. [61] reported a simple and effective interface engineering method for achieving highly efficient planar perovskite solar cells (PSCs), employing SnO_2_ electron selective layers (ESLs). A 3-aminopropyltriethoxysilane (APTES) self-assembled monolayer (SAM) was used to modify the SnO_2_ ESL–perovskite layer interface (see Figure 3). The APTES SAM demonstrated multiple functions: (1) It increased the surface energy and enhanced the affinity of the SnO_2_ ESL, which induced the formation of high-quality perovskite films with a better morphology and enhanced crystallinity. (2) The terminal functional groups formed dipoles on the SnO_2_ surface, leading to a decreased work function of SnO_2_ and an enlarged built-in potential of SnO_2_/perovskite heterojunctions. (3) The terminal groups passivated the trap states at the perovskite surface via hydrogen bonding. (4) The thin insulating layer at the interface hindered electron back transfer and reduced the recombination process at the interface effectively. These results suggest that using an ESL–perovskite interface engineered with APTES SAM is a promising method for fabricating efficient and hysteresis-less PSCs.

The use of nanostructured materials can also enhance rates of charge transport and separation in photochemical cells. These materials have a high surface area and can provide short pathways for charge transport, leading to reduced recombination losses [62]. Examples of nanostructured materials employed in photochemical cells include mesoporous metal oxides, such as TiO_2_ and ZnO, and graphene-based materials [63,64]. In particular, the incorporation of graphene into dye-sensitized solar cells has been shown to improve electron transport and reduce recombination, resulting in enhanced cell performance [65].

**Figure 4:**
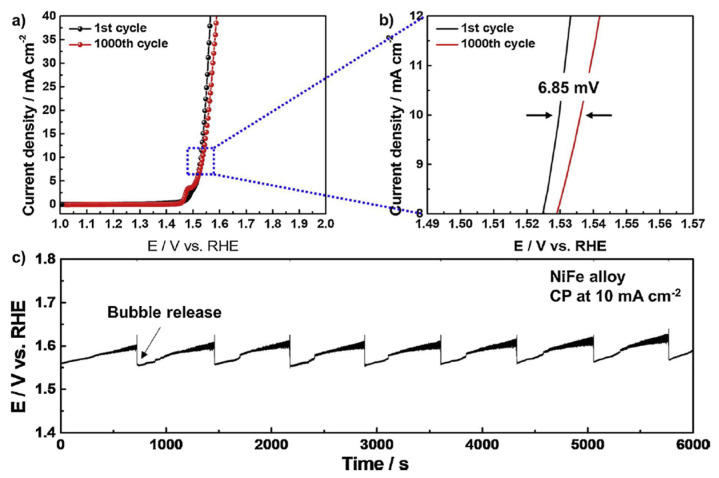
(**a**) Comparison of polarization curves of NiFe alloy between 1st and 1000th CV cycles in N_2_−saturated 1 M KOH at a scan rate of 5 mV s^−1^. (**b**) The corresponding magnified curve from 1.49 to 1.57 V versus an RHE. (**c**) Chronopotentiometry curves of the NiFe alloy in 1 M KOH at 1.65 V versus an RHE at a current density of 10 mA cm^−2^. (Reprinted with permission from Ref. [70], Copyright 2020, Catalysis Today-Elsevier).

Bimetallic catalysts have gained significant interest in recent years as a result of their potential for improved catalytic activity and stability compared to their monometallic counterparts [66]. These catalysts often exhibit synergistic effects, where the combination of two metals results in enhanced performance compared to the individual metals alone. Bimetallic catalysts have been applied to various photochemical cell systems, including dye-sensitized solar cells and water-splitting devices [67,68,69]. For example, Lim et al. [70] reported the use of a bimetallic NiFe-based alloy for oxygen evolution in a photochemical water-splitting system, which demonstrated improved catalytic activity and stability compared to the monometallic Ni and Fe catalysts. Moreover, the alloy catalyst exhibited substantial long-term durability after 1000 cyclic voltammetry tests. This electrochemical performance mainly originated from the synergistic effects of Fe incorporation into Ni species, leading to the improved charge transfer kinetics and intrinsic activity of the catalyst (see Figure 4). 

Another approach for enhancing the catalytic activity and stability of photochemical cells is the use of co-catalysts. Co-catalysts can work in synergy with a primary catalyst, promoting the desired reaction and improving the overall performance of the system. For instance, the introduction of co-catalysts such as Pt, Au, or Pd in semiconductor photocatalysts has been shown to improve the efficiency of photocatalytic water splitting by enhancing hydrogen evolution and reducing charge recombination [71,72,73]. In dye-sensitized solar cells, the use of co-catalysts, such as NiO or CuCrO_2_, can improve the performance of the system by facilitating hole transport and reducing recombination loss [74,75].

**Figure 5:**
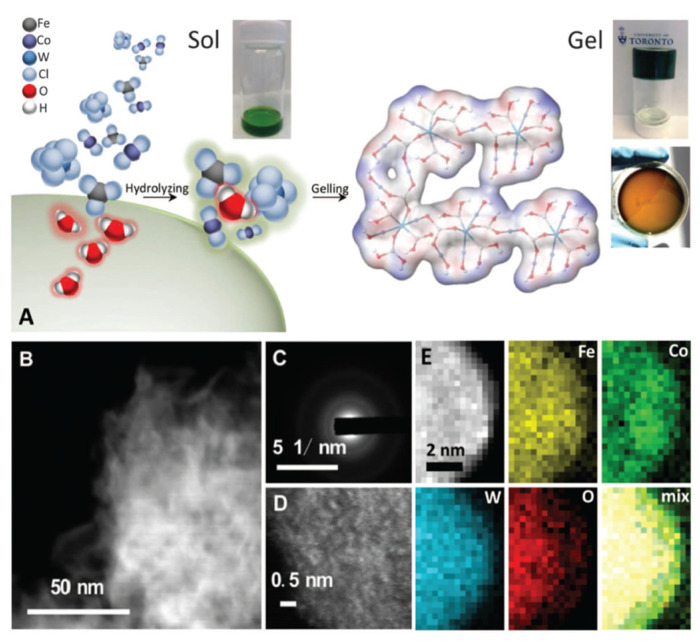
Preparation of G-FeCoWoxyhydroxides catalysts. (**A**) Schematic illustration of the preparation process for the gelled structure and pictures of the corresponding sol-gel and gelled film. (**B**) HAADFSTEM image of nanoporous structure of G-FeCoW. (**C**) SAED pattern. (**D**) Atomic-resolution HAADFSTEM image. (**E**) EELS elemental mapping from the G-FeCoW oxyhydroxide sample. (Reprinted with permission from Ref. [78], Copyright 2016, Science).

The surface modification of catalysts is another strategy for enhancing their activity and stability in photochemical cells. This can be achieved by introducing functional groups or molecules onto the catalyst surface, which can alter its electronic properties and promote the desired reactions [76]. Surface modification can also improve the stability of catalysts by providing a protective layer against degradation [77]. For example, Zhang et al. [78] developed a room-temperature synthesis to produce gelled oxyhydroxide materials with an atomically homogeneous metal distribution (see Figure 5). These gelled FeCoW oxyhydroxides exhibited the lowest overpotential (191 millivolts) and were reported at 10 milliamperes per square centimeter in an alkaline electrolyte. The catalyst showed no evidence of degradation after more than 500 h of operation. X-ray absorption and computational studies revealed a synergistic interplay between tungsten, iron, and cobalt in producing a favorable local coordination environment and electronic structure that enhance the energetics for an OER. Similarly, the surface modification of TiO_2_ with organic molecules has been shown to improve the performance of dye-sensitized solar cells by enhancing electron transfers between the dye and the semiconductor [79].

#### 2.2.2. Challenges of Photochemical Cell Performance in Artificial Photosynthesis

One of the primary challenges in the development and implementation of photochemical cells for artificial photosynthesis is scalability. Although many laboratory-scale systems have demonstrated promising results, transitioning these technologies to a large scale remains a significant hurdle [80]. The scalability challenge is multifaceted, involving the need for efficient and cost-effective production methods, the integration of photochemical cells into existing infrastructure, and the development of large-scale, stable systems that can maintain high performance over extended periods of time [81]. Overcoming these challenges is crucial for the widespread adoption and commercialization of artificial photosynthesis technologies.

Another critical challenge is the durability and stability of photochemical cells. Many of the materials and components currently used in these systems, such as organic dyes, molecular catalysts, and redox mediators, can suffer from degradation and loss of performance over time as a result of various factors, such as photobleaching, chemical instability, and mechanical stress [82,83]. Developing materials and systems that can withstand the harsh operating conditions associated with artificial photosynthesis, including high light intensities, elevated temperatures, and corrosive electrolytes, is essential for the long-term success of these technologies [84].

The cost and resource efficiency of artificial photosynthesis technologies is another significant challenge that must be addressed for widespread implementation. Many of the materials and processes currently used in photochemical cells, such as noble metal catalysts and complex fabrication techniques, can be expensive and resource intensive [85]. To make these technologies economically viable and reduce their environmental impact, it is crucial to develop more cost-effective and sustainable materials and production methods [86]. This may involve the exploration of earth-abundant alternatives to scarce and expensive materials, as well as the development of more efficient and scalable fabrication techniques [87].

In addition to the technological challenges, the environmental and social implications of artificial photosynthesis must also be considered. Although these technologies have the potential to reduce greenhouse gas emissions and contribute to a more sustainable energy future, their large-scale deployment could have unintended consequences. For example, the production of photochemical cells and their associated infrastructure may consume significant amounts of energy, water, and other resources, leading to potential trade-offs between the benefits and the environmental costs [88]. Moreover, the social implications of artificial photosynthesis, such as potential job displacement in traditional energy sectors and the equitable distribution of benefits, must be carefully considered and addressed [89].

#### 2.2.3. Strategies for Enhancing Photochemical Cell Performance in Artificial Photosynthesis

Despite the numerous challenges associated with artificial photosynthesis, there are many exciting research directions and opportunities to explore. One promising area of research is the development of novel materials and architectures that can significantly improve the performance and stability of photochemical cells. For instance, research into perovskite materials, two-dimensional materials, and metal–organic frameworks has shown great potential for enhancing light absorption, charge transport, and catalytic activity in these systems [90,91,92]. Another important research direction is the integration of artificial photosynthesis technologies with other renewable energy systems, such as solar cells, batteries, and fuel cells, to create more efficient and sustainable energy systems [93]. Furthermore, advances in computational modeling and materials informatics can help accelerate the discovery and optimization of new materials and systems for artificial photosynthesis [94]. These approaches can provide valuable insights into the fundamental mechanisms underlying the performance of photochemical cells and guide the design of more effective materials and architectures [95]. Finally, interdisciplinary collaborations between researchers in chemistry, materials science, engineering, and other fields can foster the development of innovative solutions to the many challenges facing artificial photosynthesis and contribute to the realization of its full potential as a sustainable energy technology [96].

**Figure 6:**
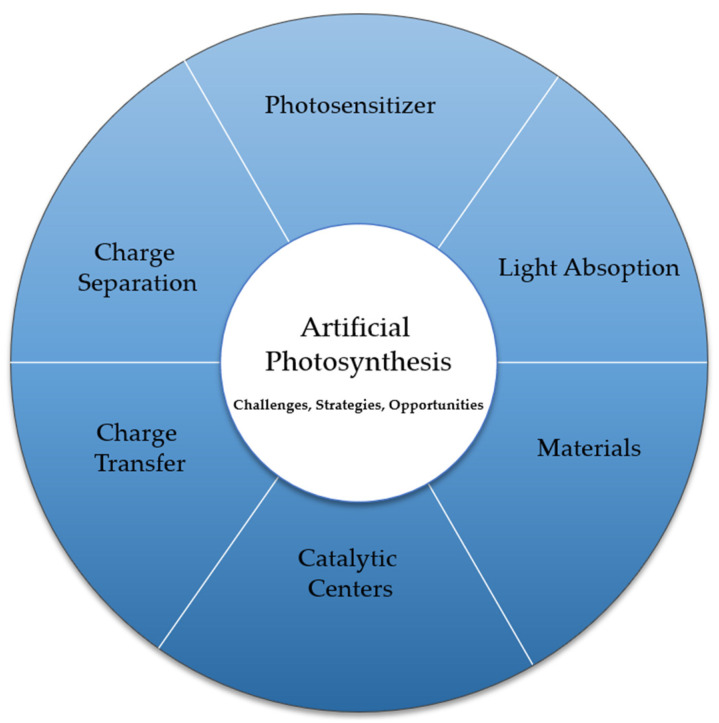
Schematic of the challenges, strategies, and opportunities of photoelectrochemical cell performance in artificial photosynthesis.

Figure 6 presents a schematic of the challenges, strategies, and opportunities of photoelectrochemical cell performance in artificial photosynthesis. Overall, the strategies, challenges, and opportunities associated with photochemical cells in artificial photosynthesis require interdisciplinary research efforts, combining materials science, catalysis, engineering, and energy policy to overcome the technical barriers and unlock their full potential for a sustainable energy future.

## 3. Hydrogen and Oxygen Evolution Reactions

Water splitting is a crucial process in artificial photosynthesis that involves the splitting of water molecules into hydrogen and oxygen using sunlight [97]. This process is essential for the production of hydrogen fuel and other value-added products using renewable energy sources. The overall reaction for water splitting can be represented as follows:

2H_2_O + photons → 2H_2_ + O_2_ (1)

**Figure 7:**
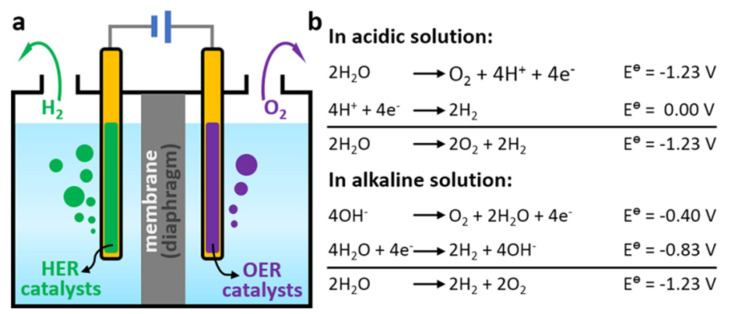
(**a**) Scheme of conventional water electrolyzers. (**b**) Water−splitting reactions under acidic and alkaline conditions. (Reprinted with permission from Ref. [97], Copyright 2018, American Chemical Society).

The process of water splitting involves two half-reactions: a hydrogen evolution reaction (HER) and an oxygen evolution reaction (OER) (see Figure 7). During an HER, protons and electrons are transferred to produce hydrogen gas, whereas during an OER, water is oxidized to produce oxygen gas and protons. Both reactions are catalyzed using a semiconductor material, typically a metal oxide such as titanium dioxide (TiO_2_), which absorbs photons from sunlight and generates electron–hole pairs that can participate in the redox reactions [33].

Efficient water splitting requires the development of catalysts that can facilitate the transfer of electrons and protons during an HER and OER. Several types of catalysts have been developed for water splitting, including metal-based and non-metal-based catalysts [98]. 

### 3.1. Hydrogen Evolution Reaction (HER)

An HER is an electrochemical process in which water is reduced to hydrogen gas. A crucial aspect of HERs is proton-coupled electron transfer (PCET), which involves the transfer of electrons from the catalyst to protons in water, eventually forming molecular hydrogen [99]. PCET is a critical component of understanding the kinetics and thermodynamics of HER, as it provides insights into the process’ reaction mechanism and energy landscape. Recent studies have focused on elucidating the PCET process in HERs using a combination of experimental and computational techniques [100,101].

An HER can be described as three elementary steps: the Volmer step, the Heyrovsky step, and the Tafel step. The Volmer step involves the electrochemical reduction of water to form adsorbed hydrogen atoms (H_ads_) and hydroxide ions (OH^−^) as follows:

H_2_O + e^−^ → H_ads_ + OH^−^ (2)

The Heyrovsky step is the electrochemical desorption of H_ads_, resulting in the formation of molecular hydrogen:

H_ads_ + H_2_O + e^−^ → H_2_ + OH^−^ (3)

Lastly, the Tafel step is the recombination of two adsorbed hydrogen atoms to produce molecular hydrogen:

2 H_ads_ → H_2_ (4)

The rate-determining step of an HER varies depending on the catalyst and conditions. Understanding the relative contributions of each step allows for the development of more efficient and selective catalysts for HERs. To develop effective HER catalysts, several criteria must be considered, including catalytic activity, stability, selectivity, and cost [102,103,104]. The ideal catalyst should have a high intrinsic activity, which is typically measured using the exchange current density and overpotential. Additionally, the catalyst should be stable under operational conditions, resistant to corrosion, and selective for the production of hydrogen. Lastly, the catalyst should be abundant, environmentally friendly, and cost effective to facilitate large-scale implementation [102,103,104].

**Figure 8:**
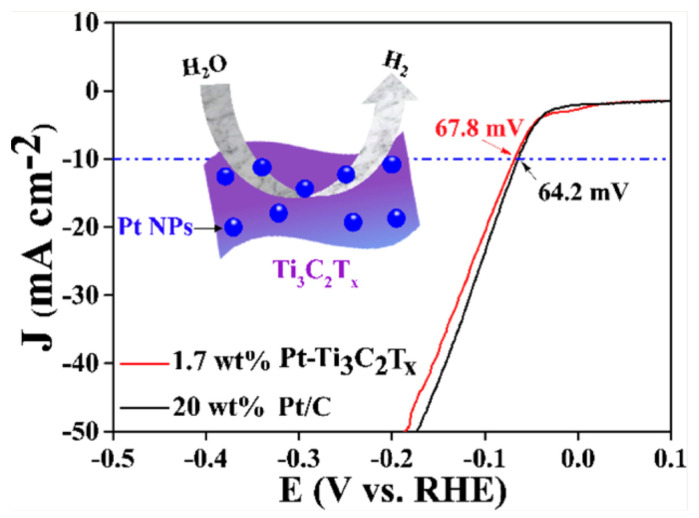
HER catalytic activity and stability showed by Pt nanoparticle (NP) −deposited 2D Ti_3_C_2_T_x_ MXenes with relatively low Pt contents (0.98−3.10 wt%). The electrochemical results indicate that the prepared catalysts showed optimal HER activity as the cycle reached 40, with an overpotential of 67.8 mV approaching that of the commercial Pt/C catalyst (64.2 mV). (Reprinted with permission from Ref. [105], Copyright 2020, American Chemical Society).

Noble metals, particularly platinum (Pt), have been widely studied as HER catalysts as a result of their high catalytic activity and stability. Zhang and group [105] prepared a series of Pt nanoparticle (NP)-deposited 2D Ti_3_C_2_T_x_ MXenes with relatively low Pt contents (0.98–3.10 wt%) that showed excellent HER catalytic activity and stability using an atomic layer deposition (ALD) method. The electrochemical results indicated that the prepared catalysts showed optimal HER activity as the ALD deposition cycle reached 40, with an overpotential of 67.8 mV approaching that of the commercial Pt/C catalyst (64.2 mV) (see Figure 8). The authors explained that the behavior was attributed to the homogeneous dispersion of the Pt NPs and the good conductivity of the 2D Ti_3_C_2_T_x_ MXene supports. However, the scarcity, high cost, and environmental concerns related to Pt limit their widespread use in large-scale hydrogen production. As a result, researchers have been exploring alternative materials, such as non-noble metal-based catalysts, to overcome these limitations [106,107].

**Figure 9:**
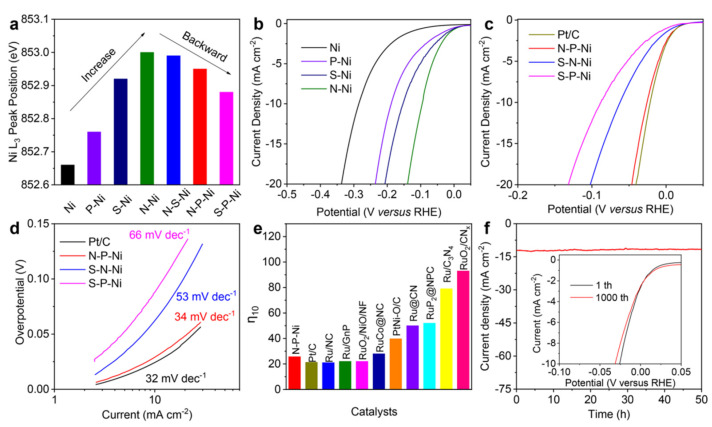
(**a**) Synchrotron-based Ni L_3_ edge peak position of single-doped and dual-doped Ni catalysts. (**b**) LSV curves of the single−doped Ni catalysts. (**c**,**d**) LSV curves and Tafel plot of the dual−doped N−P−Ni, S−P−Ni, S−N−Ni, and Pt/C. (**e**) Comparison of N−P−Ni (η10) with other noble metal-based catalysts. (**f**) Chronoamperometric curves and LSV (inset) of N−P−Ni in a long-term stability test. (Reprinted with permission from Ref. [106], Copyright 2019, American Chemical Society).

Non-noble metal-based catalysts have attracted significant attention as promising alternatives to noble metal catalysts in HERs. These materials include transition metal chalcogenides (e.g., MoS_2_, WS_2_), nitrides (e.g., MoN, VN), and phosphides (e.g., Ni_2_P, CoP) [107]. These catalysts have shown promising activity and stability, making them attractive candidates for HERs. Recent advances have focused on optimizing their electronic and structural properties to further enhance their performance [106]. For example, Jin et al. [106] reported the use of a multifaceted heteroatom doping method (nitrogen, sulfur, and phosphorus) to fine-tune the electronic structure and HER activity of non-noble metals directly and continuously without changing their chemical composition. The authors argued that doping-induced charge redistribution in the Ni metal significantly influenced its catalytic performance for an HER in alkaline media, which was confirmed by merging theoretical calculations with synchrotron-based spectroscopy, as presented in Figure 9. 

Nanostructuring and surface engineering have also been implemented to improve the performance of HER catalysts. Nanostructured materials provide a high surface area, which can increase the number of active sites, enhance rates of mass transport, and improve overall catalytic activity [108], whereas surface engineering can modify the electronic structure and facilitate the adsorption/desorption of reaction intermediates, thus improving the reaction kinetics. Numerous studies have demonstrated the advantages of nanostructuring and surface engineering in enhancing the performance of both noble and non-noble metal-based HER catalysts [108,109,110]. A research group [108] reported on surface-engineered PtNi-O nanoparticles with enriched NiO/PtNi interfaces on their surfaces. The authors explained that PtNi-O/C showed a mass activity of 7.23 mA/µg at an overpotential of 70 mV, which was 7.9 times higher compared to that of the commercial Pt/C. Meanwhile, the prepared PtNi-O/C nanostructures demonstrated significantly improved stability as well as a high current performance, both of which were well over those of the commercial Pt/C and demonstrated the capability of scaled hydrogen generation.

Heterogeneous and hybrid catalysts combine the advantageous properties of different materials to improve HER performance [111]. For example, incorporating a highly conductive material, such as graphene or carbon nanotubes, can enhance the electrical conductivity and charge transfers within the catalyst, resulting in improved activity [111]. Similarly, combining two or more catalysts with complementary properties, such as high activity and stability, can create a synergistic effect that enhances the overall performance of the hybrid catalyst [112]. Recent studies have demonstrated the potential of heterogeneous and hybrid catalysts for achieving high HER performance with a lower cost and environmental impact [111,112]. An example of this can be found in the work reported by Sun and group [112], where they demonstrated that three-dimensional NiCoSe_2_ nanosheet arrays supported on Ni foam are effective as conductive scaffolds for enhancing the catalytic activity of layered MoS_1.5_ Se_0.5_ particles. The authors explained that the resulting hierarchical MoS_1.5_ Se_0.5_/NiCoSe_2_ hybrid electrocatalyst was efficient for hydrogen evolution in acid, yielding geometric current densities of 10, 50, and 100 mA cm^−2^ at overpotentials as low as 57, 88, and 102 mV, respectively, with good long-term durability at current densities of up to 500 mA cm^−2^ over 25 h.

Rational design and computational screening have emerged as powerful tools for discovering and optimizing HER catalysts. Density functional theory (DFT) calculations and machine learning algorithms can be used to predict the properties of materials, such as adsorption energies and reaction barriers, which are crucial for understanding their catalytic performance [113]. Lu and group [113] reported that, through density functional theory (DFT) calculations, the HER activity over a 26 single-atom anchored phosphorus carbide (PC_3_) monolayer (TM@PC_3_) was studied. The results indicate that the ΔG_*H_ values of V, Fe, Nb, Mo, and Pd@PC3 were lower than those of the Pt (111) catalyst at 0.03, −0.03, −0.07, −0.04, and −0.02 eV, respectively. Machine learning (ML) was employed to explore the intrinsic relationship between catalytic performance and feature parameters. The authors demonstrated that the first ionization energy, bond length of TM-H, and d band center are more correlated with hydrogen adsorption behavior. Their work predicted that Fe, Nb, and Mo@PC_3_ can be substitutes for Pt metal in HER and, also, revealed the intrinsic correlation between catalytic activity and feature parameters by combining DFT and ML investigations. 

### 3.2. Oxygen Evolution Reaction (OER)

An oxygen evolution reaction (OER) is another crucial half-reaction in water splitting that involves the four-electron oxidation of water to produce oxygen. The reaction can be summarized as follows:

2H_2_O → O_2_ + 4H^+^ + 4e^−^ (5)

Because of its multi-electron nature, an OER is inherently more complex and energetically demanding than a hydrogen evolution reaction (HER) [114]. The need for four protons and four electrons in an OER significantly contributes to its kinetic sluggishness and high overpotential [114]. Understanding the key intermediate states and reaction steps of OERs is vital to the design and optimization of efficient OER catalysts for artificial photosynthesis. An OER generally proceeds via the so-called “OER cycle,” which involves the formation and deprotonation of various hydroxylated intermediates on the catalyst surface [114]. The OER cycle typically starts with the adsorption of a water molecule on the catalyst surface, followed by its deprotonation to form an adsorbed hydroxyl group. This hydroxyl group is then further deprotonated and oxidized to form a higher oxidation state intermediate, which eventually undergoes O–O bond formation to produce an oxygen molecule [114]. The exact nature and structure of these intermediates, as well as the rate-determining step of the OER cycle, can vary depending on the specific catalyst and reaction conditions.

The interactions between the catalyst surface and the OER intermediates play a crucial role in determining the catalytic activity of an OER. These interactions can be quantified in terms of the adsorption energies of the intermediates, which significantly influence the reaction barriers and rate-determining step [115]. The optimization of the surface interactions and adsorption energies is a major challenge in the design of OER catalysts. Ideally, a catalyst should bind the intermediates neither too weakly nor too strongly, a concept referred to as the “Sabatier principle” [115]. Achieving this balance is critical for facilitating the sequential deprotonation and oxidation steps while avoiding over-stabilization of the intermediates, which can lead to catalyst deactivation [115]. Furthermore, the catalyst surface can also interact with the protons and electrons involved in the OER, influencing the proton-coupled electron transfer (PCET) processes that are essential for the OER. The catalyst’s electronic structure, as well as its geometric and electronic interactions with the adsorbed intermediates, can significantly affect the PCET kinetics and the overall OER activity as well [115].

**Figure 10:**
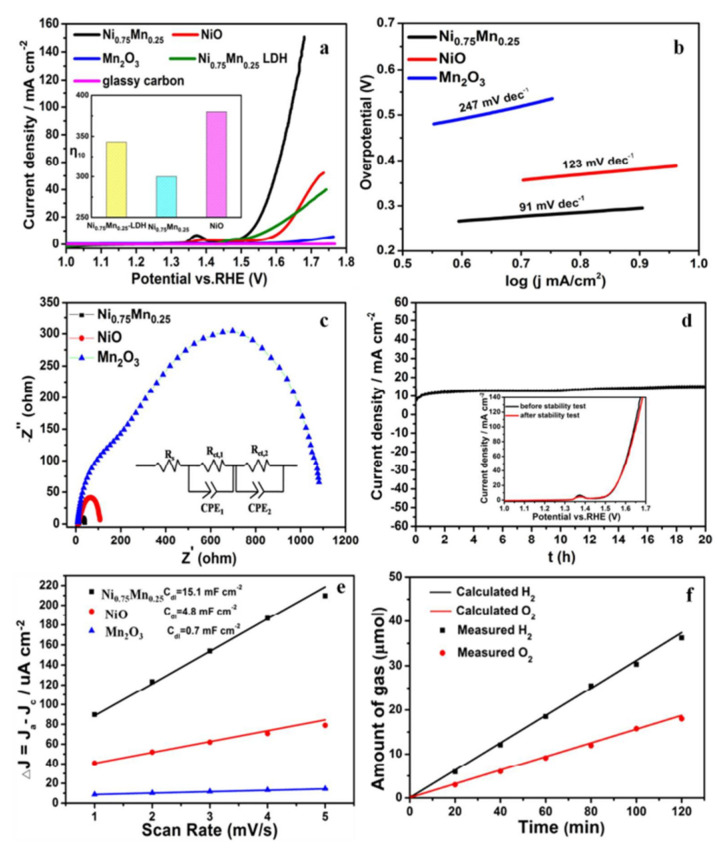
(**a**) LSV curves of Ni_0.75_ Mn_0.25_−LDH, Ni_0.75_ Mn_0.25_, NiO, Mn_2_O_3_, and glassy carbon (inset shows the η10 of Ni_0.75_Mn_0.25_-LDH, Ni_0.75_Mn_0.25_, and NiO), (**b**) Tafel slopes, and (**c**) Nyquist plots of Ni_0.75_ Mn_0.25_, NiO, and Mn_2_O_3_ at an applied potential of 1.65 V. (**d**) Chronoamperometric durability test for the Ni_0.75_ Mn_0.25_ at a constant current density of ~10 mA cm^−^^2^ (inset shows the corresponding polarization curves before and after the stability test). (**e**) Charging current density differences (∆J = J_a_ – J_c_) plotted against scan rates. (**f**) Amount of experimental and theoretical O_2_ and H_2_ evolution by Ni_0.75_ Mn_0.25_ at a constant oxidative current of 1 mA. (Reprinted with permission from Ref. [116], Copyright 2018, American Chemical Society).

Engineering catalysts at the nanoscale can significantly enhance their performance by increasing their surface area and number of active sites available for an OER [116]. For example, nanostructured cobalt oxides, manganese oxides, and nickel oxides have shown improved OER activity as a result of their high surface-to-volume ratios and abundance of catalytically active sites [116,117]. A study conducted by Tian and group [116] investigated the active site of nickel oxide nanosheets using manganese modulation in an electrocatalytic oxygen evolution system. The authors explained that the electronic structure could be realized via Mn modulation, and that the intrinsic catalytic activity of Ni^3+^ (t_2g_^6^e_g_^1^) and Jahn–Teller active Mn^3+^ (t_2g_^3^e_g_^1^) species act synergistically to promote an electrocatalytic oxygen evolution reaction. Furthermore, an X-ray absorption near-edge structure analysis indicated that the Ni^3+^ and Mn^3+^ in Ni_0.75_Mn_0.25_ nanosheets may result from nickel vacancies and oxygen vacancies, thus resulting in higher oxygen evolution activity than is found in NiO and Mn_2_O_3_ (see Figure 10).

As mentioned, the kinetics of an OER can be significantly affected by the rate of charge transfer and transport on the catalyst surface. Nanostructuring can improve these processes by reducing the length scales for charge transport and by optimizing the catalyst’s electronic structure [118,119]. Moreover, the surface of the catalyst can be engineered by doping or by creating hybrid structures with conductive materials, which can enhance the electrical conductivity and facilitate the charge transfer and transport processes involved in an OER [118]. A study conducted by Ding et al. [118] explained that, because of the excellent corrosion resistance of the Fe–Co–Ni–Cr–Nb high entropy intermetallic Laves phase, fabricating a high entropy bulk porous nanostructure is possible by dealloying the corresponding eutectic alloy precursor. The authors argued that a core–shell nanostructure with amorphous, ultrathin, high entropy oxide films wrapped around nanosized intermetallic ligaments was obtained, together exhibiting an extraordinarily large active surface area, fast dynamics, and long-term durability and outperforming the existing alloy- and ceramic-based OER electrocatalysts. 

As was seen for HERs, heterogeneous and hybrid catalysts can also exploit the synergistic effects between different materials to improve the OER performance [120]. For instance, hybrid catalysts composed of a transition metal oxide and a conductive carbon material can benefit from the high OER activity of the metal oxide and the excellent conductivity of the carbon material [120]. Similarly, heterogeneous catalysts composed of multiple metal oxides can leverage the different catalytic properties of each metal oxide to optimize the OER performance [121]. An example of this can be seen in the work composed by Tariq and group [121], where they reported the development of an OER-beneficial mixed oxide composite of molybdenum and iridium oxides using a hydrothermal method. The authors explained that IrO_2_ nanoparticles that adhered synergistically to large MoO_3_ particles possessed a more robust nature toward harsh acidic water electrolysis than toward electrolysis in an alkaline environment. The results showed that the mass specific OER activity of iridium active centers was enhanced sevenfold, was twice the current density, and was attributed to the electronic modulation of noble metal. Furthermore, the authors argued that an enhanced surface area and the existence of highly oxidative species in the O(1s) spectrum of IrO_2_ and two doublet regions in the X-ray photoelectron spectrum of molybdenum metal were found, accounting for the robust performance.

**Figure 11:**
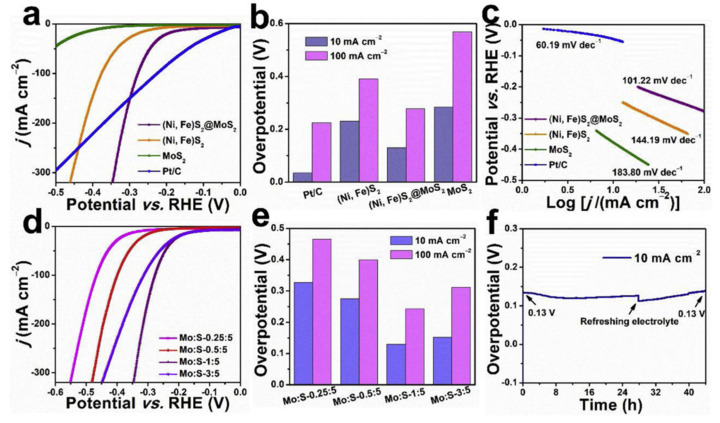
(**a**) LSV curves, (**b**) overpotentials at current densities of 10 mA cm^− 2^ and 100 mA cm^− 2^, and (**c**) Tafel slopes of (Ni, Fe)S_2_@MoS_2_, (Ni, Fe)S_2_, MoS_2_ and Pt/C. (**d**) LSV curves and (**e**) overpotential at 10 mA cm^− 2^ and 100 mA cm^− 2^ of (Ni, Fe)S_2_@MoS_2_ (Mo:S—0.25:5, 0.5:5, 1:5 and 3:5). (**f**) Electrochemical stability test at 10 mA cm^− 2^ of (Ni, Fe)S_2_@MoS_2_. (Reprinted with permission from Ref. [123], Copyright 2019, Applied Catalysis B: Environmental).

The design and optimization of the interfaces between different materials in a heterogeneous or hybrid catalyst are crucial for maximizing their OER performance. Proper interface engineering can enhance charge transfer rates across the interface, stabilize the catalyst structure, and even create additional active sites for OERs [122,123]. For example, in a hybrid catalyst composed of a metal oxide and a carbon material, the metal–carbon interface can be engineered to facilitate a charge transfer from the metal oxide to the carbon material, thereby improving the overall OER performance [123]. Metal chalcogenides, such as MoS_2_, have also been employed to optimize interfaces for OER reactions. For example, Liu and group [123] employed an interface engineering strategy to construct a bifunctional electrocatalyst based on (Ni, Fe)S_2_@MoS_2_ heterostructrues for a water-splitting process. The results show (see Figure 11) that the as-prepared (Ni, Fe)S_2_@MoS_2_ catalyst exhibited good electrochemical activity and durability under alkaline environments, with a low overpotential of 270 mV for an OER to deliver the current density of 10 mA cm^−2^. The authors further explained that, in combination with an in-situ Raman spectra, the constructed interfacial active sites were favorable for the formation of S-H_ads_, which synergistically lowers the chemisorption energy of the intermediates of HERs and OERs, thereby facilitating the overall electrocatalytic water splitting. 

As was seen for HERs, DFT calculations and machine learning have also become a powerful tool for the rational design of OER catalysts. They allow for the prediction of the electronic structure, adsorption energies, and reaction barriers of different catalyst materials and structures, thereby guiding the design of catalysts with optimal properties for an OER [124,125]. DFT calculations have been used to predict the OER performance of various transition metal oxides, perovskites, and layered double hydroxides, among other materials [125], whereas machine learning can identify patterns and correlations in the screening data to predict the OER performance of untested catalysts [124]. Deng and group [125] used DFT coupled with machine learning technology to explore the structure–property correlation and catalytic activity origin of bi atoms catalysts (BACs), where metal dimers were coordinated using N-doped graphene (NC). They sampled 26 homonuclear (M_2_/NC) BACs and constructed an activity volcano curve; however, only one BAC, namely Co_2_/NC, exhibited promising ORR activity. Then, they extended the study to 55 heteronuclear BACs (M_1_M_2_/NC) and found that 8 BACs possessed competitive or superior ORR activity compared with the Pt(111) benchmark catalyst. Linear scaling relationships among the adsorption free energy levels of *OOH, *O, and *OH species were significantly weakened on BACs as compared to on a transition metal surface, indicating that it was difficult to precisely describe the catalytic activity with only one descriptor. Thus, they employed machine-learning techniques to identify the origins of ORR activity on BACs, which is mainly governed by simple geometric parameters. The authors argued that their work identified promising BACs and provides useful guidelines for the development of novel and highly efficient ORR catalysts.

## 4. Catalytic Carbon Dioxide Reduction

**Figure 12:**
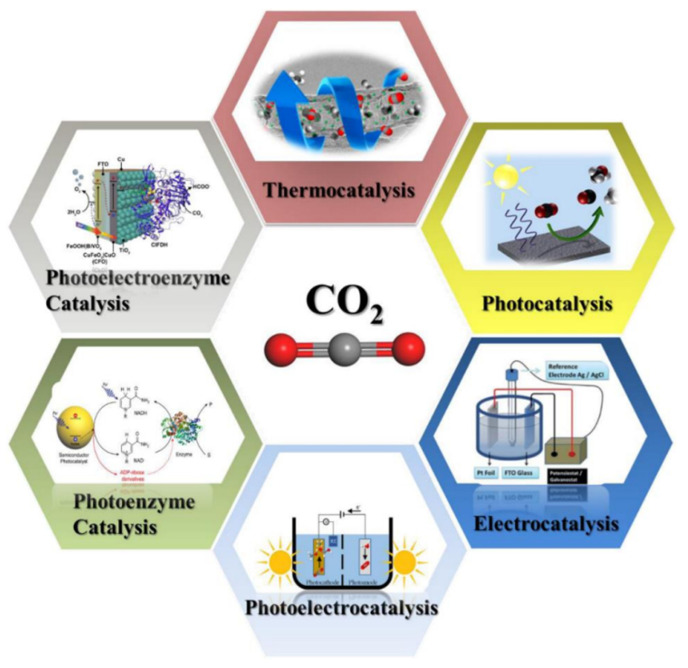
Schematic showing the strategies that are usually used in CO_2_ conversion. Reprinted with permission from Ref. [127], Copyright 2020, Molecules-MDPI.

**Figure 13:**
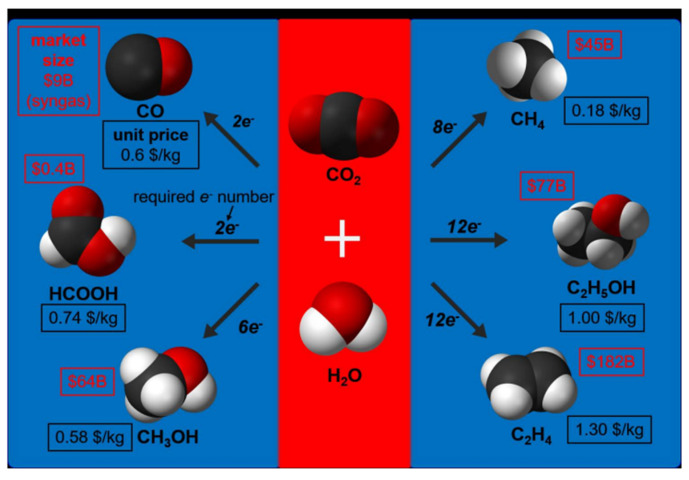
Various possible routes for the products of a CO_2_ reduction reaction. The market size, unit price, and required number of electrons for producing each chemical are marked in the schematic. Reprinted with permission from Ref. [128], Copyright 2019, Catalysts—MDPI.

This process involves the use of specialized materials to convert CO_2_ into hydrocarbons, which can be used as a fuel. This approach has the potential to significantly reduce greenhouse gas emissions by utilizing CO_2_ as a raw material. This conversion can be achieved through various methods, such as electrocatalysis, photocatalysis, photoelectric catalysis, and biocatalysis (see Figure 12). Some of the possible routes for the products of a CO_2_ reduction reaction are presented in Figure 13. However, these processes are complex as a result of some significant limitations. Among these limitations, it should be noted that, under ambient temperature and pressure conditions, the solubility of CO_2_ in water is only 0.033 M [126], causing it to perform poorly when competing with water molecules during the adsorption process. The low diffusion rate and solubility of CO_2_ in aqueous solutions greatly limit the efficiency of CO_2_ conversion. Moreover, CO_2_ is a nonpolar linear molecule with two strong bonds, which necessitates a high use of energy to break the C-O bonds, resulting in low conversion rates [127]. Considering all of these limitations, processes have been under study for years to mitigate them and improve the efficiency and selectivity of CO_2_ conversion. The aim is to develop systems capable of mimicking nature to reduce atmospheric CO_2_ levels and utilize this carbon in the synthesis of high-value compounds.

### 4.1. Electrocatalytic Approach

Electrocatalysis has gained extensive attention because of its benefits, including mild reaction conditions, a flexible product adjustment, low yields of byproducts, and the utilization of renewable energy sources. The electrochemical reduction of CO_2_ to chemicals is viewed as a sustainable method to combat global warming while promoting economic growth. However, the current market lacks commercially available technologies for a CO_2_ electroreduction reaction [128]. Achieving cost-competitive CO_2_ electrolysis necessitates meeting specific criteria, such as a high current density, high selectivity, or even long-term operation. Current density plays a crucial role in evaluating catalytic performance, as it reflects the reaction rate. Significant technical improvements are required to overcome these limitations in the development of new electrocatalysts, electrolyzers, and electrolytes and in all aspects of managing such processes.

Formate is a valuable fuel generated from CO_2_ reduction reactions that is useful for direct electricity generation in fuel cells [129]. Heavy-metal-based catalysts, such as tin, indium, lead, or bismuth, among others [130,131,132,133], are commonly used for formate synthesis, whereas copper-based catalysts [134] are typically preferred for producing hydrocarbons and alcohols of various nature that require subsequent separation treatments. However, these CO_2_ reduction processes compete to some extent with other reactions, such as catalytic water splitting, to produce hydrogen. Therefore, these processes require significant improvement to enable their practical application. In this regard, Dai et al. [135] synthesized Cu_2_O/Cu films that exhibited a high efficiency for the electroreduction of CO_2_, up to 98%, with the formation of practically pure formate. The authors further observed that the efficiency of CO_2_ electroreduction was dependent on the purity of the copper utilized in the fabrication process [135]. Additionally, they also identified a distinct correlation between an elevated CO_2_ pressure and an increased selectivity towards formate, thereby suggesting a substantial enhancement in process efficiency and an evident reduction in associated costs [135]. Sargent et al. [136] discovered that high-local electric fields, which lead to a higher local concentration of CO_2_ near the active CO_2_ reduction reaction surface at low applied overpotentials, are generated by nanostructured electrodes. In their study, various experiments were conducted by the authors using gold nanoneedles, confirming that the field-induced reagent concentration enabled the CO_2_ reduction reaction to proceed with superior performance, surpassing previous results obtained with various gold structures and noble metal oxides [136]. Additionally, it was demonstrated in their research that palladium nanoneedle electrocatalysts allowed for the highly efficient synthesis of formate. The efficiency of palladium-based materials was also demonstrated in the work by Chen et al. [137], who showed that different facets of Pd influence its performance in CO_2_ reduction. In their study, nanosized Pd cubes and octahedra particles, mainly with (100) and (111) facets, were synthesized, and it was found that Pd octahedra particles exhibited a higher selectivity and better activity than Pd cubes or commercial particles. The investigation identified the formation of PdH during the reduction reaction, and an interaction between different intermediates and PdH was found to be one of the relevant factors in improving the efficiency and selectivity of the process [137]. Han et al. [138] employed N,P-co-doped carbon aerogels in a CO_2_ reduction to CO for the first time. The outcomes manifested a notable efficacy of the process with a partial current density of −143.6 mA cm^−2^, representing one of the highest current densities to date. The catalysts manufactured in the investigation showcased a superior electrochemical active area and conductivity compared to those of N- or P-doped carbon aerogels, thereby augmenting the electron transfer from CO_2_ to its radical anion or other intermediates involved in the process [138]. Moreover, the research confirmed the pyridinic N species’ high activity for CO_2_ reduction to CO, and the co-doping of P with N was found to be crucial in impeding a hydrogen evolution reaction (HER) [138]. In a separate study, Liu et al. [139] utilized nitrogenated graphene to disperse nickel and create a durable and efficient electrocatalyst for CO_2_ reduction. The results of the study showed that the catalytically active site was the monovalent Ni(I) atomic center with a d9 electronic configuration. The single-Ni-atom catalyst exhibited a high level of activity, and even after more than 100 h of continuous use, the activity remained at approximately 98% of its initial value [139].

In addition to the diverse types of catalysts previously showcased, it is noteworthy to highlight processes involving molecular catalysis. Metal complexes that coordinate with conjugated ligands exhibit improved electrocatalytic properties capable of carrying out CO_2_ reduction. To this end, catalysts based on metal-containing phthalocyanines or porphyrins have been developed, which have demonstrated considerable efficacy for this process [140,141,142]. In this regard, Qiu et al. [143] synthesized two novel electrocatalysts for CO_2_ reduction by employing dimethoxy-substituted Co(II) porphyrin. The authors suggested that the methoxy group’s electron donor effect facilitated the intramolecular charge transfer, thereby promoting electron mobility. Furthermore, theoretical calculations using density functional theory (DFT) confirmed that the methoxy group could substantially affect the energy levels of the process, thereby enhancing CO_2_ reduction efficiency [143]. In another study, Shi et al. [144] synthesized a hybrid cobalt(II) phthalocyanine-COOH/carbon nanotube (CNT) catalyst by anchoring CoPc-COOH onto the surface of carbon nanotubes. This particular catalyst displayed an enhanced performance compared to other CoPc-COOH/CNT and CoPc/CNT catalysts. The excellent performance of the catalyst was credited to the charge transfer phenomenon caused by the inclusion of -COOH and -NH_2_ functional groups in both CoPc and CNT, which expedited the formation of active Co(I) centers at lower potentials. As a result, the catalyst displayed the highest turnover frequency (TOF) and achieved optimal efficiency [144]. 

In another recent study, the design and synthesis of an Fe porphyrin catalyst with an appended N,N-di(2-picolyl)ethylenediamine (DPEN) unit was described, which exhibited enhanced electrocatalytic activity in CO_2_ reduction reactions when water was used as the proton source. The DPEN unit captures water molecules and facilitates proton transfer while also stabilizing negatively charged CO_2_ reduction intermediates. These factors enable this catalyst to achieve a high electrocatalytic CO_2_ reduction activity with a remarkable TOF of 5.0 × 10^4^ s^−1^.

### 4.2. Photocatalytic Approach

Photocatalytic CO_2_ reduction processes, based on an artificial photosynthetic scheme, necessitate the involvement of a photosensitizer that captures sunlight and delivers a high-energy electron to a catalytic center, being ultimately responsible for CO_2_ reduction [145]. Homogeneous and heterogeneous studies have been conducted for many years with the aim of improving this process. Although significant advancements have been made, the practical applicability of current systems still falls short of expectations. In this context, it is worth noting some of the notable breakthroughs achieved in recent years.

As with many catalytic processes, metal-based photosensitizers have shown great activity in CO_2_ photoreduction. Among these catalysts, one developed by Xiong et al. [146] based on a Ni-terpyridine complex can be mentioned. During the CO_2_ photoreduction reaction, a reduced Ni(I) intermediate is produced, which is coordinated with a terpyridine ligand, CO_2_, and solvent molecules (acetonitrile) to generate a penta-coordinated species that ultimately enables CO_2_ reduction to CO. In another study, a copper purpurin complex was developed as a catalyst [147]. This complex contained an additional redox-active center, which enabled it to shift the reduction potential to 540 mV less than that of its organic dye component. When this copper photosensitizer was combined with an iron porphyrin catalyst and sacrificial reductant, the system achieved a remarkable turnover number (TON) of 16,100 for the reduction of CO_2_ to CO, with a very high selectivity for CO [147]. 

**Figure 14:**
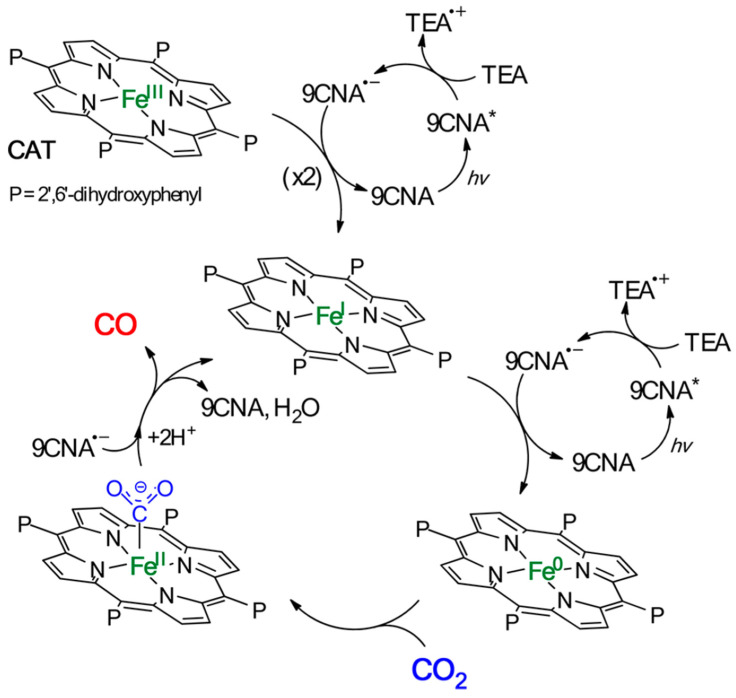
Proposed mechanism for the catalytic reduction of CO_2_ to CO using an iron porphyrin catalyst (CAT) in the presence of a photosensitizer (9CNA) and a sacrificial electron donor (TEA). (Reprinted with permission from Ref. [151], Copyright 2014, ACS).

**Figure 15:**
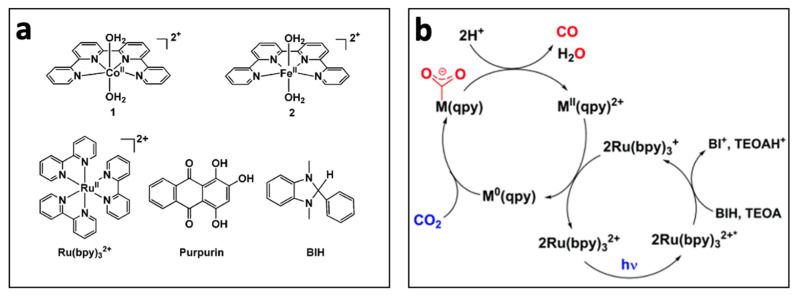
Structures of the catalysts, photosensitizers (Ru(bpy)_3_^2+^, Purpurin), and sacrificial reductant, BIH (**a**) and the proposed mechanism for the photochemical reduction of CO_2_ to CO (**b**). (Reprinted with permission from Ref. [152], Copyright 2016, ACS).

**Figure 16:**
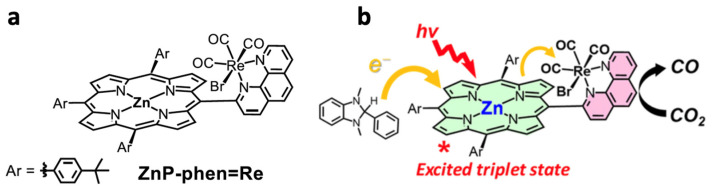
Structure of the porphyrin–rhenium complex (ZnP-phen = Re) (**a**) and the proposed mechanism for the photocatalytic chemical reduction of CO_2_ to CO (**b**). (Reprinted with permission from Ref. [153], Copyright 2020, American Chemical Society).

Organic photosensitizers are an interesting option for photocatalytic CO_2_ reduction [148]. However, this alternative presents some challenges, such as the fact that only a few of these compounds absorb radiation in the visible range, greatly limiting their application [149]. Furthermore, efficiency is another limitation, as it requires the use of high concentrations of these compounds for the reaction to proceed under acceptable conditions [150]. In this context, some catalysts have emerged that show remarkable behavior. Bonin et al. [151] used an iron-based homogeneous catalyst (iron porphyrin) and a photosensitizer that was exposed to visible light. They discovered that the system did not degrade and produced CO with high selectivity for 50 h. The outcomes were especially noteworthy when utilizing 9-cyanoanthracene (9CNA) as a photosensitizer in the presence of triethylamine (TEA), which acted as a sacrificial electron donor, attaining TONs of approximately 60 and a catalytic selectivity for CO of 100% [151]. This is an impressive result for a system that uses an iron-based catalyst, an inexpensive organic sensitizer, and, especially, can operate with visible light. Figure 14 depicts the scheme proposed by the authors for this photocatalyst. In another investigation [152], two catalysts, [Co(qpy)(OH_2_)_2_]^2+^ and [Fe(qpy)(OH_2_)_2_]^2+^, were employed (see Figure 15). It was observed that, under visible-light excitation using Ru(bpy)_3_^2+^ as the photosensitizer and 1,3-dimethyl-2-phenyl-2,3-dihydro-1H-benzo[d]imidazole (BIH) as the sacrificial reductant, the cobalt catalyst achieved a high turnover number (TON) of 2660 with a 98% selectivity for CO [152]. Similarly, the iron catalyst achieved a TON of over 3000 with an approximately 95% selectivity. Furthermore, by replacing Ru(bpy)_3_^2+^ with the organic dye sensitizer purpurin, which is more accessible and inexpensive, the cobalt and iron catalysts attained TONs of 790 and 1365, respectively, in N,N-dimethylformamide [152]. In a separate study, Satake et al. [153] synthesized a porphyrin–rhenium complex (ZnP-phen = Re) consisting of a zinc porphyrin photosensitizer directly linked to a rhenium complex (see Figure 16). The ZnP-phen = Re system efficiently captured and stored light energy in the form of a long-lived T_1_ state of the zinc porphyrin. At room temperature and in the absence of BIH and oxygen, T_1_ phosphorescence was detected. When ZnP-phen = Re was used in the presence of BIH and phenol, a nearly complete photocatalytic reduction of CO_2_ to CO was achieved without any catalyst decomposition [153]. The remarkable stability of the system, with a minimum of 1300 TON_CO_, was attributed to a strong interaction between the rhenium and porphyrin components, which served as an electron reservoir to prevent electron accumulation on the porphyrin and the resulting hydrogenation of its skeleton [153].

In a recent study, Han et al. [154] elucidated the application of a series of uncomplicated organic light-absorbing agents, composed of anthraquinone dyes, for promoting the reduction of CO_2_ using visible light. The authors demonstrated high activity for both the PS and the catalyst. Their mechanistic investigation proposed that the hydroxyanthrone forms of PS (PSH_2_ and PSH_2_^−^), which are produced from reductive quenching, are significant intermediates in the light-driven catalytic steps [154]. The most effective PS was identified by introducing both electron-donating and electron-withdrawing groups to the anthraquinone. 

### 4.3. Photoelectrochemical (PEC) Approach

PEC offers a greener method of producing fuels and chemicals from CO_2_ under light irradiation. It combines the advantages of electrocatalytic and photocatalytic approaches while improving the separation efficiency of photogenerated electron–hole pairs [155]. The generation of energy-rich chemical feedstocks, such as ethylene (C_2_H_4_), is useful for a range of applications [156]. In addition, alcohols such as methanol (CH_3_OH) are essential for hydrogen storage and are utilized in the production of gasoline and biodiesel [157]. Depending on the type of electrodes used in PEC cells, we can distinguish different photoreactor configurations: (i) dark anode/photocathode system [158]; (ii) photoanode/dark cathode [159]; and (iii) photoanode/photocathode [160]. In any case, the selection of the photoelectrodes and electrolyte is crucial for the performance of the process. There are numerous examples of different systems and their efficiency, but one common factor is the use of n-type semiconductors as photoanodes and p-type semiconductors as photocathodes [161]. When light is directed at a photoanode, it generates pairs of electrons and holes. The electrons are then transferred towards the cathode, where they participate in reduction reactions, while the holes contribute to oxidation reactions on the photoanode’s surface. Conversely, in the case of a photocathode, electron–hole pairs are generated after exposure to light, and the photogenerated electrons participate in reduction reactions on the surface of the photocathode [161]. At the same time, oxidation reactions take place on the photoanode’s surface as a result of the holes produced by the light. When both photoanodes and photocathodes are exposed to light, photogenerated electrons in the photoanode are transported to the photocathode through an external bias potential and combined with the electrons produced by the light in the photocathode. Meanwhile, the holes produced in the photoanode contribute to oxidation reactions in the electrolyte of the photoanode compartment [161]. 

**Figure 17:**
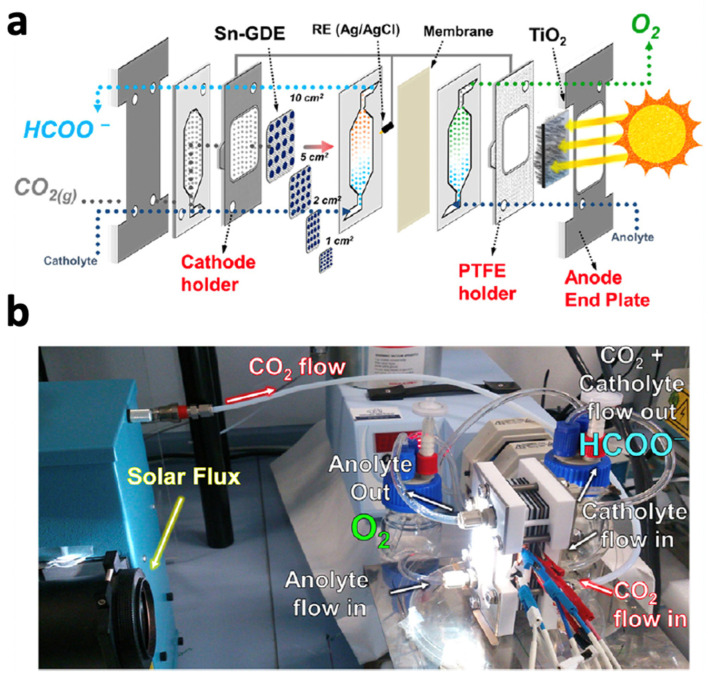
Scheme of the photoelectrochemical flow cell using TiO_2_ photoanode (**a**) and photograph of the PEC system setup (**b**). (Reprinted with permission from Ref. [166], Copyright 2017, Elsevier).

Some p-type photocathodes include materials such as GaP, CuO, CdTe, or InP, among others, which can be highly unstable or even toxic in a solution [162]. As an alternative, n-type semiconductors, such as ZnO [163], Fe_2_O_3_ [164], and WO_3_ [165], among others, which are much more stable, significantly less toxic, and can be easily obtained, have been integrated into PEC systems. However, one of the most studied materials, as a result of its low cost, low toxicity, and ease of preparation, is TiO_2_, which has been used as a photoanode. Nevertheless, TiO_2_ presents several drawbacks for its practical use in PEC systems. Andreu et al. [166] fabricated a PEC system utilizing TiO_2_ nanorods and an electrodeposited Sn on a gas diffusion electrode (GDE) for the conversion of CO_2_ to HCOO^−^ under continuous flow conditions (Figure 17). Through the optimization of the PEC cell and the coupling of both electrodes, Sn-coated GDE carbon fibers exhibited superior electrocatalytic activity for CO_2_ reduction, surpassing the inherent limitations of the photoanode. At a potential of 0.95 V, the system demonstrated faradaic efficiencies greater than 40%, resulting in a solar-to-fuel efficiency of 0.24% after optimization [166]. In another study, Kim et al. [167] presented a study on the use of reduced Ag catalysts on TiO_2_/p-Si photocathodes for CO_2_ reduction to produce syngas. A patterned SiO_2_ layer was introduced to allow for light absorption and facilitate CO_2_ reduction on Ag catalysts. The best-performing photocathode had a low onset potential of −0.16 V vs. RHE and a high saturated photocurrent density of −9 mA/cm^2^ at −1.23 V vs. RHE [167]. A faradaic efficiency of 47% for CO was achieved at −0.6 V vs. RHE, producing a syngas ratio of 1:1 at a rate of 18.6 mol/s∙cm^2^, suitable for the Fischer–Tropsch synthesis [167]. 

**Figure 18:**
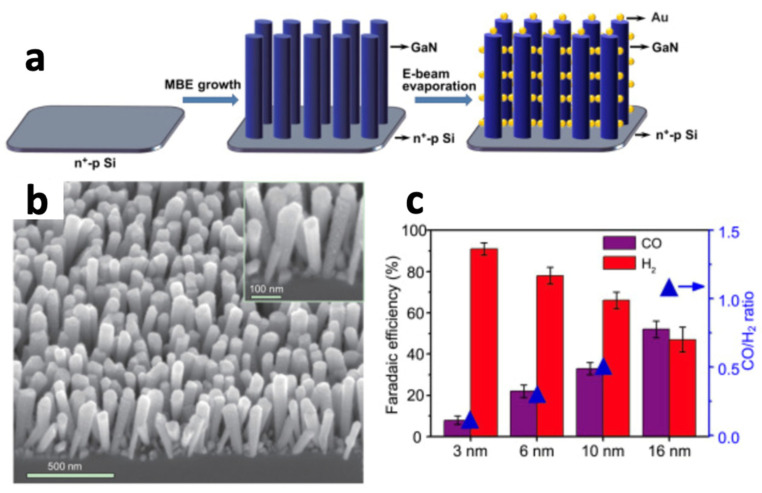
Fabrication procedure of Au/GaN/n^+^-p Si sample scheme (**a**); SEM image of the grown heterostructure (**b**); and the correlation between the faradaic efficiency of the process and the diameter of the gold nanoparticles on the material (**c**). (Reprinted with permission from Ref. [168], Copyright 2022, Elsevier).

Chu et al. [168] developed a catalytic system based on Au/TiO_2_ with GaN/n^+^-p Si (Figure 18), which demonstrated efficient and controllable PEC syngas generation through CO_2_ reduction. The integrated photocathode achieved a solar energy conversion efficiency of 2.3%. Additionally, desirable CO/H_2_ ratios of syngas compositions, such as 1:2 and 1:1, were obtained simply by adjusting the particle size of Au nanoparticles dispersed on the TiO_2_ semiconductor, which is a promising result that will allow for the modulation of the final process outcome through catalyst engineering.

Ruotolo et al. [169] synthesized two copper vanadates with different morphological characteristics and distinct properties. The type of semiconductor composition, either n-type β-Cu_2_V_2_O_7_ or p-type α-CuVO_3_, was influenced by the copper site valence in the precursor. Although the α-CuVO_3_ photoelectrode was unstable and converted to β-Cu_2_V_2_O_7_, the latter exhibited considerable efficiency in generating methanol (ca. 236 μmol cm^−2^ h^−1^) with high selectivity [169]. Recently, Reisner and colleagues [170] developed an efficient PEC system that utilizes a single light-absorber without any applied voltage to simultaneously perform solar-driven CO_2_ reduction and plastic reformation to generate value-added products. The system integrates three different types of CO_2_ reduction catalysts, including a molecular catalyst (cobalt porphyrin), a bimetallic alloy (Cu_91_In_9_), and a biocatalyst (formate dehydrogenase), with a perovskite light absorber to form photocathodes. Additionally, a bimetallic alloy serves as an oxidation catalyst for reforming polyethylene terephthalate (PET) plastic to glycolic acid with a faradaic efficiency exceeding 90% [170]. The PEC system has a tunable product distribution with high selectivity and significant formation rates for CO, syngas, and formic acid in combination with PET reforming at the anode. Their research represents the unique demonstration of a sustainable process that combines solar-driven CO_2_ reduction with plastic waste valorization, leading to substantial improvements in both areas.

### 4.4. Biocatalytic Approach

The natural conversion of atmospheric CO_2_ into organic compounds via the Calvin cycle during the process of photosynthesis is known as carbon fixation, which occurs in plants, algae, and some bacteria. These enzymatically controlled processes are distinguished by their exceptional selectivity and specificity, as well as their remarkable efficiency and mild operational parameters [171,172,173]. The fixation of CO_2_ in aquatic algae is by far the predominant photosynthetic process in nature, producing twice as much biomass as that generated by terrestrial plants. This is facilitated by the rapid growth of these organisms and their easy access to nutrients. It is for this reason that photosynthetic algae can be considered a living laboratory that we can use to understand how these processes occur and how we can use them from a practical standpoint. In this regard, Valle et al. [174] described an improvement in the yield of an enzymatic reduction of CO_2_ to methanol using three enzymes co-immobilized in siliceous mesostructured cellular foams (MCF): (i) formate dehydrogenase (FateDH), which converts CO_2_ to formate; (ii) formaldehyde dehydrogenase (FaldDH) for the conversion of formate to formaldehyde; and (iii) alcohol dehydrogenase (ADH), which converts formaldehyde to methanol. The host silica material was functionalized to improve the enzyme–support interaction, and the enzymes were fluorescently labeled to monitor their uptake and distribution. The enzymes were immobilized, and two protein loadings were tested. The study found a 4.5-fold higher methanol yield when the enzymes were immobilized in order of increasing size and with a loading of 50 mg (enzymes) g^−1^ (support) [174]. The study suggested that, by using MCF, a simple method of immobilization can be applied to significantly increase enzyme activity for a cascade reaction. In another investigation, Reisner et al. [175] described the utilization of immobilized enzymes, specifically formate dehydrogenase, as ideal catalysts because of their high turnover and selectivity at a minimal overpotential. Their investigation explored the impact of CO_2_ hydration on the performance of CO_2_ reduction systems by studying the effect of co-immobilizing carbonic anhydrase. The results demonstrated that the co-immobilization of carbonic anhydrase enhanced the kinetics of CO_2_ hydration, thereby improving enzymatic CO_2_ reduction by reducing local pH changes. In other cases, the aim of CO_2_ fixation has been to obtain minerals or other high-value-added products. For instance, Chafik et al. [176] employed carbonic anhydrase (CA) in CO_2_ sequestration and CaCO_3_ production, which hold significant industrial applications. To achieve this, a stable and efficient CA that can tolerate high concentrations of CO_2_ and Ca^2+^, high pH, and high working temperatures is required. In their study, the authors reported on the sequestration of CO_2_ into CaCO_3_ using a novel CA purified from the liver of a camel, an animal known for its ability to survive extreme desert conditions. The enzyme, which is a monomer with a molecular mass of 25 kDa, contains Fe as a physiologically relevant cofactor instead of Zn and exhibited a higher optimum pH (pH 9.0) and temperature (45 °C), even functioning at higher temperatures (60 °C) [176]. The enzyme was found to be highly efficient in converting CO_2_ to CaCO_3_ (966.67 mg CaCO_3_/mg enzyme) in the presence of high concentrations of Ca^2+^ [176]. In a separate investigation [177], the isolation of bacterial CA from *Corynebacterium flavescens* was carried out. In this case, the purified CA exhibited an optimal temperature of 35 °C and pH 7.5 and was found to be majorly inhibited by Na^+^, K^+^, Mn^2+^, and Al^3+^ ions, whereas Zn^2+^ and Fe^2+^ ions slightly enhanced its activity. The purified CA demonstrated a notable efficacy in the conversion of CO_2_ into CaCO_3_, with a total conversion rate of 65.05 mg CaCO_3_/mg of protein [177]. Another enzyme that is used in CO_2_ fixation processes is Ribulose-1,5-bisphosphate carboxylase/oxygenase (RuBisCO) [178], which participates in the Calvin–Benson–Bassham Cycle (CBBC). This reaction is complex and consists of five partial reactions that result in the production of two molecules of 3-phosphoglycerate. This enzyme plays a relevant role in biological CO_2_ assimilation and, in one study, was expressed in *Escherichia coli*, allowing for CO_2_ to be co-metabolized with glucose to produce metabolites [179]. However, RuBisCO is known for its low k_cat_ and for forming inhibited complexes with its substrate ribulose-1,5-bisphosphate and other sugar phosphates. In the study, RuBisCO forms I and II were cloned and expressed in Escherichia coli for in situ CO_2_ capture. The findings reveal that both form I and form II RuBisCO exhibit similar activities in E. coli and result in comparable levels of in situ CO_2_ recycling. In very recent research, Liu et al. [180] conducted a study in which they engineered RuBisCO form 1A from the proteobacterium *Halothiobacillus neapolitanus* in *Escherichia coli* and tobacco chloroplasts. They replaced the native tobacco gene that encodes the Rubisco large subunit with *H. neapolitanus* RuBisCO (HnRuBisCO) large and small subunit genes. The study showed that HnRubisco subunits were able to form functional hexadecamers effectively in tobacco chloroplasts. These hexadecamers had a carboxylation rate that was approximately two times higher than that of the wild-type and supported a growth rate of transgenic plants that was similar to that of the wild-type in air supplemented with 1% CO_2_. This is a significant advancement towards the bioengineering of RuBisCO to enhance CO_2_ capture processes. Phosphoenolpyruvate carboxylase (PEPC) is another enzyme that participates in the CO_2_ fixation process. Plants possess a natural mechanism to concentrate CO_2_ near RuBisCo to promote carboxylation and suppress photorespiration. During photosynthesis, carbonic anhydrase (CA) converts atmospheric CO_2_ into HCO_3_^−^, which is utilized by PEPC to synthesize C_4_ acids. In another study, Molla et al. [181] isolated the PEPC gene from the plant *Setaria italica* and transferred it to rice. An overexpression of SiPEPC increased the enzyme activity by 2–6 times in transgenic lines compared to the non-transformed control. Transforming plants enhanced their photosynthetic efficiency, leading to an increase in the PSII quantum yield and higher chlorophyll accumulation. Additionally, an increased PEPC activity improved the quantum yield and carboxylation efficiency, leading to an increase in the yield and biomass of transgenic PEPC lines by ca. 23–29% and 24–29%, respectively [181]. These findings imply that increasing the expression of the specific PEPC enzyme has the potential to enhance photosynthesis, making it a promising avenue for future developments. 

**Figure 19:**
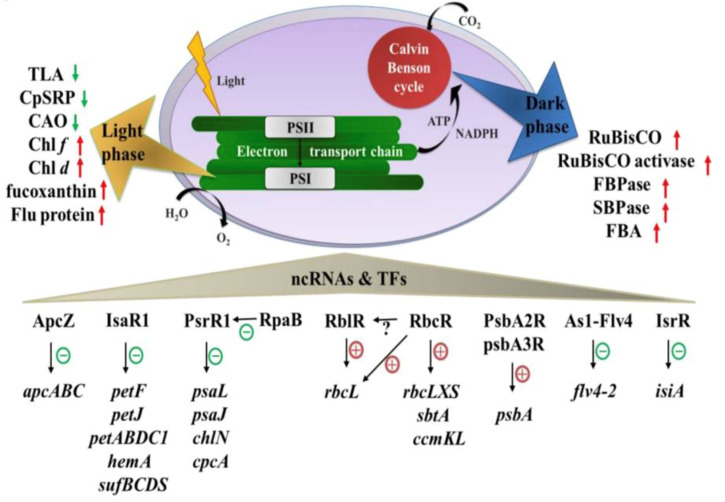
Potential options for enhancing photosynthesis in microalgae include overexpression or introduction of exogenous genes (depicted as red arrows), or down-regulation or knockout of certain genes (shown as green arrows), which may have a promoting effect (indicated by red crosses) or a hindering effect (indicated by green minus signs). (Reprinted with permission from Ref. [182], Copyright 2023, MDPI).

The results of these studies have inspired several research projects aimed at enhancing CO_2_ capture for both environmental and economic purposes (Figure 19) [182]. Photosynthesis, in general, is an inefficient process, with over 75% of the radiation that reaches plants and algae being lost [183]. Limitations in the electron transport chain and the photosystems responsible for it contribute to this inefficiency [184]. Various strategies, including crop improvement, the induction and selection of mutants showing higher efficiencies, and genetic engineering processes capable of modifying the peptide structure of key enzymes, particularly their active centers, are being developed, particularly in algae. Mutations related to RuBisCO, the rate-limiting enzyme in photosynthesis, are of particular interest as they have the potential to improve CO_2_ capture efficiency [185]. Developing more efficient RuBisCO isomorphs is the primary option for substantially enhancing the process.

## 5. Biomimetic Approaches

Enzymes are regenerable catalytic machineries developed by nature. Many of these enzymes outperform synthetic catalysts when it comes to selectivity, rate, and energy efficiency, particularly in complex chemical transformations that occur in mild aqueous conditions. The reason behind this is that the protein scaffold and microenvironment surrounding the active site of enzymes restrict the conformation of substrates and high-energy intermediates, thereby controlling the outcome of reactions [186,187,188,189,190,191]. One remarkable example of such a reaction is the H_2_O-oxidation reaction, which is both kinetically and thermodynamically demanding. This reaction represents the initial step in the process of photosynthesis, which is initiated in photosystem II (PS-II) and catalyzes the oxidation of water at +1.2 V vs. a standard hydrogen electrode (SHE) that utilizes a Mn_4_O_5_Ca oxygen-evolving complex, to generate electrons [192]. Photosystem I (PS-I), in its part, generates the largest reduction potential found in nature (−1.2 V), which is utilized in the production of ATP and NADH, as well as in carbon fixation in photosynthetic organisms. Autotrophic and heterotrophic organisms express different types of enzymes, such as hydrogenases, carbon monoxide dehydrogenase, or nitrogenases, which can be involved in relevant catalytic processes, such as hydrogen production, CO_2_ reduction and fixation, and N_2_ fixation [193,194,195]. Unlike synthetic catalysts, whose activity is very limited, these enzymes can participate in catalytic processes with turnover frequencies of more than 10,000 s^−1^ and with extraordinary selectivity. A paradigmatic example of this type of enzymes is the cytochrome c oxidase (CcO), which has developed over millions of years in nature and, thanks to the presence of certain metals, such as Fe, Cu, Ni, or Mn, is responsible for the fixation of CO_2_ and N_2_ in the construction of essential compounds for life [196]. The extraordinary activity of these natural catalysts is due to the perfect organization of the active center and the ideal disposition of the rest of the enzyme’s structure, generating layers around the active center that allow for the efficient transfer of electrons and reaction intermediates to or from the active center [197]. These examples, found in nature, have been used in attempts to create bioinspired or hybrid materials that allow for much more efficient processes with high selectivity. However, enzymes are very sensitive to the physicochemical conditions of the environment, being effective in very narrow ranges of pH, temperature, ionic strength, and solvent. In some cases, these limitations have been overcome through the development of hybrid structures formed by compounds of a natural origin that have been supported on inorganic structures, generating hybrid materials capable of withstanding conditions very different from those used in nature.

### 5.1. Biohybrids for Enzymatic Catalysis

In the past several years, organic chemists have been using visible light photoredox catalysis as a technique to carry out synthetic organic transformations, inspired by the photosynthesis process [198,199]. This method utilizes metal complexes and organic dyes to initiate single-electron transfer processes with organic substrates upon photoexcitation with visible light. This technique can also be used to initiate photopolymerization reactions using free radical or cationic mechanisms [200]. In this regard, Guo et al. [201] developed an eco-friendly method to produce tetrahydroquinolines from N,N-dimethylanilines and maleimides. The approach is highly sustainable as it employs visible light, oxygen, and chlorophyll, which acts as the photosensitizer. 

Yang et al. reported a metal-free approach to synthesizing amides from thioamides. This method employs chlorophyll as a photosensitizer to generate singlet molecular oxygen, ^1^O_2_, which is involved in the aerobic desulfurization of thioamides. Gajewska et al. [202] used copper trisodium chlorophyllin as an efficient catalyst for atom transfer radical polymerizations of poly(ethylene glycol) acrylate (PEGA) in PEGA-chlorophyllin copolymers with a controlled content of chlorophyllin, suitable for drug delivery, biomedical materials, and solar energy harvesting [202]. 

In the classic works by Moore et al. [203,204] the use of artificial reaction centers based on complex structures formed by the covalent incorporation of porphyrin, naphthoquinone, and carotenoid embedded in a liposomal bilayer is described. In their research, light absorption resulted in acidification of the interior of the liposomal structure. Under these conditions, an ATP synthase catalyzed the reaction of ATP formation as a result of the proton gradient present on both sides of the liposomal membrane. 

Another example of the synthesis of compounds of interest mediated by naturally occurring enzymes is demonstrated by Gandomkar et al. [205]. In their research, they utilized the enzyme cytochrome P450, obtained from *Clostridium acetobutylicum,* for the conversion of saturated fatty acids to α-ketoacids. In this reaction, they were able to recycle the oxidant H_2_O_2_, thereby minimizing degradation of the ketoacid product and maximizing the biocatalyst’s lifetime. In other research, Park et al. [206] presented a photoelectrochemical device composed of a FeOOH/BiVO_4_ photoanode, a Cu(In,Ga)Se_2_ solar absorber, and a graphitic carbon/reduced graphene oxide cathode for carrying out light-driven peroxygenase catalysis. The designed system was capable of producing H_2_O_2_, which, in the presence of *Agrocybe aegerita* peroxygenase, facilitated the stereoselective hydroxylation of ethylbenzene, leading to the development of (R)-1-phenylethanol with a remarkable enantioselectivity of over 99%.

### 5.2. Bacteriorhodopsins

**Figure 20:**
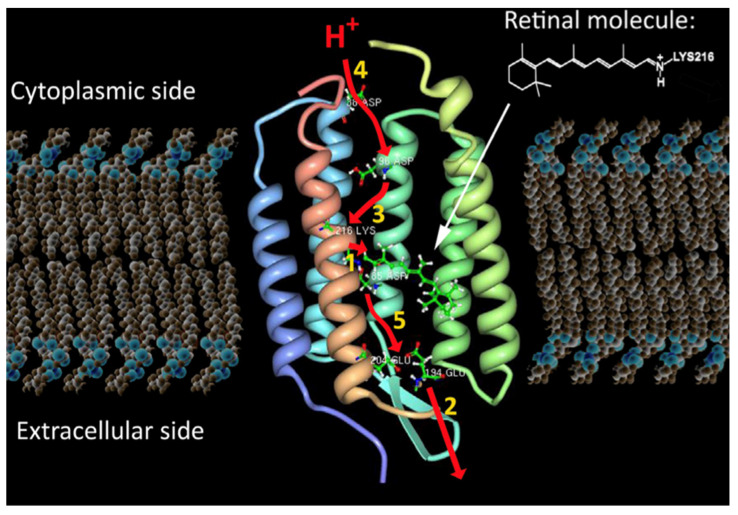
bR embedded in a bilipid membrane, consisting of seven transmembrane α-helices, with one helix removed for clarity, and a chromophore called retinal. The red arrows and the corresponding numbers in yellow show the sequence of protonation events that occur during the transport of a proton from the cytoplasmic to extracellular side of the membrane. (Reprinted with permission from Ref. [208], Copyright 2014, Elsevier).

Another example of a molecule found in nature that is capable of using solar radiation is bacteriorhodopsins (bRs) [207], which are a set of different proteins found in the cell membranes of certain bacteria, such as *Halobacterium salinarum*, as well as in Eukaryotes and Archaea. The bRs from *Halobacterium salinarum* consist of seven transmembrane α-helices (see Figure 20). Every bR molecule possesses a chromophore known as retinal that is covalently attached to lysine-216 in the G helix via a protonated Schiff base, which is located at the center of a cavity that is enclosed by the seven transmembrane helices [208]. It effectively splits the proton channel into two parts, namely the extracellular and cytoplasmic half-channels. bRs are unique in that they are capable of converting the energy of green light (500–650 nm) into a transmembrane proton gradient, which can then be used to generate ATP [209]. This process, known as the bR cycle, involves the absorption of a photon of light by the protein’s retinal chromophore, causing a conformational change that pumps a proton from the cytoplasmic side of the membrane to the extracellular side [209]. Various types of bRs have been employed for the assembly of hybrid structures using semiconductors such as TiO_2_ [210,211] and ZnO [212]. These structures have been utilized in the development of solar cells, increasing their efficiency and representing a potential alternative for the development of energy conversion systems. bRs have also been used when functionalized with TiO_2_ gel in the presence of platinum nanoparticles for hydrogen production [213]. Under these conditions, it was observed that bRs did not denature even in the presence of ethanol or methanol, and the hydrogen production from these hybrid structures was 52% higher than that observed in the absence of bR. 

Kuruma et al. [214] developed an artificial cell system containing an artificial organelle capable of converting light energy into electrochemical potential and then into the chemical energy of ATP, used for reactions such as aminoacylation of tRNA, GTP generation, and protein translation. The system also demonstrated its ability to photosynthesize bR and ATP synthase, enhancing the activity of ATP production. The artificial cell synthesized its own part, bR, in a positive feedback loop, and the phosphate recycling system could be used in a cell-free system. This research could help reveal the transition from nonliving to living matter that occurred on early Earth. 

**Figure 21:**
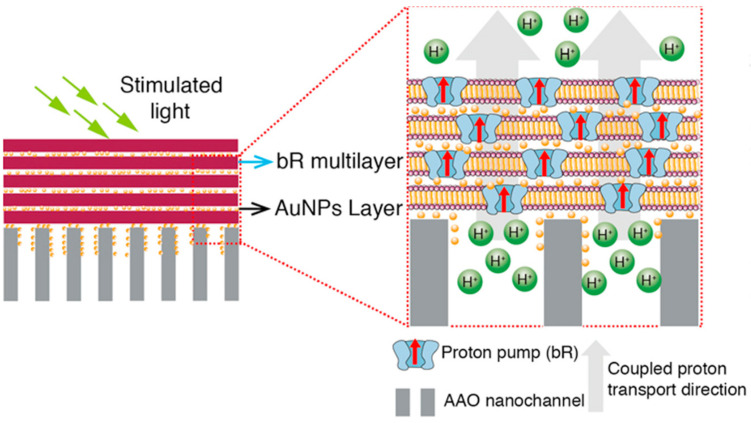
Schematic representation of the bR/AuNP heterogeneous structure. The AAO membrane was used as the substrate for alternating deposition of bR and Au nanoparticles. Upon light exposure, proton transfer occurred from the cytoplasmic side to the extracellular side. The surface plasmonic effect of AuNPs shortened the photocycle path, resulting in more efficient proton pumping within a fixed time. (Reprinted with permission from Ref. [215], Copyright 2014, Elsevier).

In another study [215], a novel photovoltaic stack system based on heterogeneous multilayers of bacteriorhodopsin (bR)/gold nanoparticles (AuNPs), mimicking the stack structure of granum, was demonstrated for the first time (Figure 21). By controlling the diameter of AuNPs and the stacking layers, a value of approximately 350 nA cm^−2^ was reached, and the photocurrent could be effectively regulated [215]. This hybrid system has the potential to be used as a solar energy converter to power nano-devices.

### 5.3. Nanohybrids for Hydrogen Production

Photosystem II (PSII) was the first photosystem to operate in light-dependent reactions. It contains several pigment–protein complexes, including the reaction center (RC) complex, which contains chlorophyll a, and the primary electron acceptor, pheophytin, as well as the light-harvesting complex (LHC) that surrounds the RC complex and contains numerous accessory pigments. When light is absorbed by the pigments in the LHC, it is funneled to the RC complex, where it excites an electron in chlorophyll a to a higher energy state. This high-energy electron is then transferred to the primary electron acceptor, pheophytin, initiating a chain of electron transfers that eventually culminate in the oxidation of water molecules to oxygen and the generation of a proton gradient across the thylakoid membrane, which is used to produce ATP. Photosystem I (PSI) operates downstream of PSII and contains a similar set of pigment–protein complexes. The RC complex of PSI contains chlorophyll a, but it is distinct from that of PSII, and the primary electron acceptor is ferredoxin. When excited by light, the electron in chlorophyll a is transferred to the primary electron acceptor and subsequently passes through a series of electron carriers, including a soluble electron carrier, namely plastocyanin, before being used to reduce NADP+ to NADPH. This electron transfer generates a proton gradient across the thylakoid membrane that is used to produce ATP via the ATP synthase.

**Figure 22:**
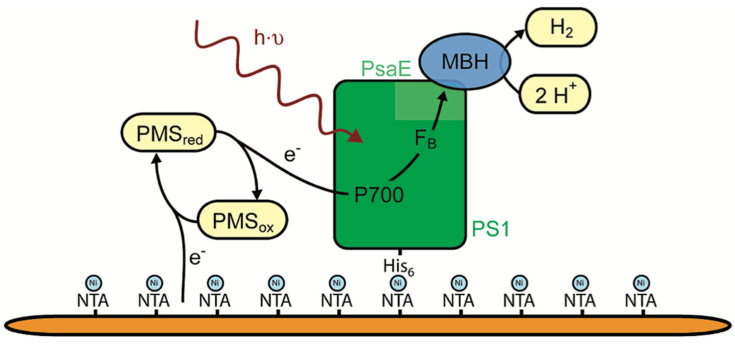
Schematic view of the nanodevice, which comprises a gold electrode that supplies electrons leading to the reduction of the oxidized form of N−methylphenazonium methyl sulfate (PMS). Subsequently, PMS donates an electron to the chlorophyll a dimer P700 in PSI, which becomes excited to a higher energy level upon exposure to light. The electrons then travel through the acceptor site’s FB cluster before being transferred to the distal iron sulfur cluster of the hydrogenase (MBH). Finally, the electrons arrive at the active site, where protons are reduced to form molecular hydrogen. (Reprinted with permission from Ref. [216], Copyright 2009, ACS).

The function of PSI and PSII is highly coordinated, and the transfer of electrons between them is crucial for maintaining electron flow and the generation of cellular energy. In this regard, Heberle et al. [216] assembled PSI and hydrogenase to achieve the efficient conversion of solar energy into hydrogen. The authors demonstrated the successful assembly on a solid gold surface (see Figure 22), producing hydrogen when exposed to light. Compared to previously described (bio) nanoelectronic devices that did not use the photosynthesis apparatus, this device can produce hydrogen at a lower energy cost. The authors suggested that this successful demonstration could provide a model for establishing this system in living organisms with the significant advantage of self-replication [216]. 

Although remarkable results have been achieved by combining hydrogenases with isolated photosystems to generate hydrogen, these systems have a limited lifespan as a result of the absence of metabolic processes that support self-repair and maintenance. The production of photosynthetic hydrogen using a PSI-hydrogenase fusion in vivo has been demonstrated by Gutekunst et al. [217]. Specifically, the NiFe-hydrogenase of the cyanobacterium *Synechocstis* sp. was fused to its PSI in close proximity to the chemical cluster responsible for donating electrons to ferredoxin. The resulting mutant was found to grow photoautotrophically and generate a high concentration of photosynthetically produced hydrogen (500 μM) under anaerobic conditions in the light without taking up the generated hydrogen [217]. In a similar manner, a novel chimeric polypeptide consisting of PSI and a hydrogenase enzyme was developed [218]. This chimera utilizes the endogenous hydrogenase of *Chlamydomonas reinhardtii*, which is more abundant and physiologically active. When expressed in a *C. reinhardtii* strain devoid of native hydrogenases, the chimera successfully formed active PSI–hydrogenase complexes, capable of repairing itself in vivo, and was able to power the Calvin–Benson–Bassham cycle, resulting in high rates of O_2_ production [218]. In this case, hydrogen production persisted for at least four days using a combination of media that significantly reduced CO_2_ fixation and an O_2_-scavenging agent.

Schuhmann et al. [219] fabricated a photocathode by immobilizing photosystem I (PSI) protein complexes in a dense and anisotropic structure that promoted efficient unidirectional electron flow. The use of redox polymers facilitated electron transfer and prevented short-circuiting processes. The photocathode was coupled with a hydrogenase for light-induced H_2_ evolution [219], and the potential for a fully light-driven water splitting cell was demonstrated.

Mann et al. [220] developed a discrete cellular micro-niche that is capable of sustained photosynthetic and photosynthetic-independent hydrogen production by interfacing living algal cells with a conductive polymer and a calcium carbonate exoskeleton. In this research, localized hypoxic conditions and hydrogenase activity were induced under daylight in air, resulting in photosynthesis-independent hydrogen evolution for up to 200 days through a direct extracellular photoelectron pathway to hydrogenase [220]. Additionally, hydrogen production was observed for up to eight days in surface-conductive dead algal cells [220]. In other research, Nagata et al. [221] described the use of PSI extracted from *Thermosynechococcus vulcanus* and combined it with Pt nanoparticles (PtNP) to achieve hydrogen production in the presence of visible light. Lumogen Red (LR), an artificial light-harvesting dye, is also utilized in this reaction system, and all three components (PSI, PtNPs, and LR) are necessary for hydrogen evolution. LR absorbed the visible light and transferred energy to PSI, resulting in charge separation at the reaction center of the PSI. Excited electrons then gathered in the PtNPs, leading to hydrogen evolution. These findings are certainly interesting because the wavelength range for the process can be expanded, increasing efficiency and potential applications, depending on the dye used. In a separate investigation [222], carbon dots (CDs) that were highly fluorescent and based on aspartic acid were synthesized and utilized as a photosensitizer for driving photocatalytic hydrogen evolution with an [FeFe] hydrogenase. The interaction between the CDs and the hydrogenase was studied to determine the impact of the electrostatic environment on the biohybrid assembly’s photocatalytic performance. The study yielded an initial activity of 1.73 μmol (H_2_) mg^−1^ (hydrogenase) min^−1^ [222]. It is noteworthy that the synthesized CDs demonstrated proficient operation under visible light and displayed stability for over a week.

### 5.4. Nanohybrids for CO_2_ Reduction

Nocera et al. [223] developed a hybrid water-splitting biosynthetic system that utilizes a biocompatible catalyst to split water at low driving voltages. *Ralstonia eutropha*, when grown in contact with the catalysts, consumed the produced hydrogen to synthesize biomass and fuels/chemicals from low concentrations of CO_2_ in the presence of O_2_ with an efficiency of ca. 50%, allowing it to scrub 180 g of CO_2_ per kilowatt-hour of electricity. The authors stated that this system allows for obtaining CO_2_ reduction efficiencies that exceed the efficiency of natural photosynthetic processes.

**Figure 23:**
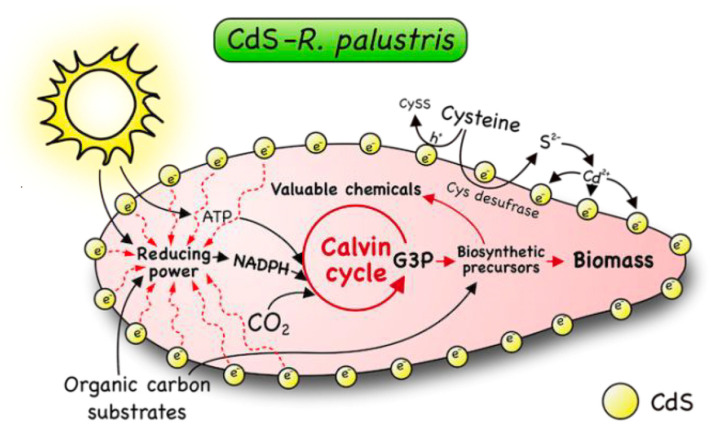
Illustration depicting the improved CO_2_ reduction and production of valuable chemicals in the hybrid system of CdS nanoparticles and R. palustris. (Reprinted with permission from Ref. [224], Copyright 2019, Royal Society of Chemistry).

Wong et al. [224] developed a hybrid system by coating CdS nanoparticles onto a versatile photosynthetic bacterium, R. palustris, resulting in efficient CO_2_ reduction and valuable C_2+_ chemical production. This hybrid system showed good performance under practical conditions and represents a truly interesting example for future real-life applications. The mechanism proposed by the authors is based on the presence of toxic Cd^2+^ ions, which trigger the release of S^2−^ from Cys by Cys desulfurase [224]. This results in the formation of insoluble CdS nanoparticles that coat the cell surface and generate photocurrents under visible light irradiation. The remaining Cys effectively eliminates the holes, leading the cell to continuously experience reducing stress that is transformed into cellular reducing equivalents (NADPH). This stimulation of the Calvin cycle releases excessive reducing equivalents by fixing more CO_2_, resulting in the capture of additional energy by the cell for biosynthesis and valuable chemical production [224] (see Figure 23).

In a separate study, Ghirlanda et al. [225] demonstrated the use of artificial protein catalysts based on cytochrome b562, incorporating cobalt protoporphyrin IX as a cofactor, for light-driven CO_2_ reduction in water under mild conditions. The incorporation of the cofactor into the protein scaffold enhanced the reactivity of the cobalt porphyrin, resulting in improved proton reduction and CO generation [225]. By modifying the binding site, the activity of the enzyme was adjusted, indicating that rational design or directed evolution could potentially enhance catalytic activity even further. These findings are consistent with previously published results [226,227] and offer a vast array of possibilities for maximizing CO_2_ reduction using biological nanohybrids with selected mutations. In a study by Yuan et al. [228], an integrated system was developed for the conversion of CO_2_ into bioplastics, utilizing a common microorganism called *Pseudomonas putida*, thus contributing to climate change mitigation through CO_2_ capture. The authors devised a novel electro-microbial CO_2_ conversion system called EMC2, which uses soluble C2 intermediates, such as acetate and ethanol, for the production of bioproducts. As suggested by the authors, the EMC2 method exhibits clear advantages over other existing CO_2_ conversion systems, including but not limited to higher reducing equivalents, superior energy and mass transfer, and the capacity to engineer a wider array of products. The study successfully tackled numerous challenges, such as catalyst selection in biocompatible electrolytes, interference of mineral cations in growth media, and low concentration ethanol distillation. EMC2 represents a paradigm shift in utilizing CO_2_ as a feedstock for producing a diverse range of commodity chemicals and products. Another interesting study by Ma et al. [229] involved the development of a chemical–biochemical hybrid pathway for synthesizing starch from CO_2_ and hydrogen in a cell-free system. This process consists of 11 core reactions that were computationally designed, subsequently assembled, and optimized through the protein engineering of three bottleneck-associated enzymes. This process results in the conversion of CO_2_ into starch at a rate of 22 nanomoles of CO_2_ per minute per milligram of the catalyst, which is approximately 8.5 times faster than the rate of starch synthesis in maize, indicating a promising potential for future advancements.

### 5.5. Biomimetic Models Anchored onto Heterogeneous Supports

Biomimetic models anchored onto heterogeneous supports, such as metal–organic frameworks (MOFs) and silica-based materials, have shown significant promise for their potential in artificial photosynthesis applications as well [230,231,232,233,234,235,236,237,238]. MOFs, with their flexible structure and high surface area, can provide an ideal environment for biomimetic complexes. These can emulate the structure and function of natural photosynthetic centers, thus facilitating the critical light-absorbing and charge-transfer processes involved in artificial photosynthesis. For instance, Pullen and group [230] developed a molecular proton reduction catalyst [FeFe]-(dcbdt)(CO)_6_ (1, dcbdt = 1,4-dicarboxylbenzene-2,3-dithiolate) with structural similarities to [FeFe]-hydrogenase active sites and incorporated it into a highly robust Zr(IV)-based metal−organic framework (MOF) via a postsynthetic exchange (PSE). The authors explained that, in conjunction with [Ru(bpy)_3_]^2+^ as a photosensitizer and ascorbate as an electron donor, MOF-[FeFe](dcbdt)(CO)_6_ catalyzes photochemical hydrogen evolution in water at pH 5. Furthermore, the group argued that the immobilized catalyst shows substantially improved initial rates and overall hydrogen production when compared to a reference system of complex 1 in solution. The improved catalytic performance was ascribed to a structural stabilization of the complex when incorporated in the MOF. Similarly, a molecular H_2_-evolving catalyst, [Fe_2_(cbdt)(CO)_6_] ([FeFe], cbdt = 3-carboxybenzene-1,2-dithiolate), developed by Roy et. al. [231], was attached covalently to an amino-functionalized MIL-101(Cr) through an amide bond. The authors explained that chemical reduction experiments revealed that MOF channels can be clogged by ion pairs that are formed between the oxidized reductant and the reduced catalyst. This effect was lessened in MIL-101-NH-[FeFe] with lower [FeFe] loadings, meaning that on longer timescales, the proportions of the [FeFe] catalysts within the MOF engage in photochemical hydrogen production. Additionally, the amount of produced hydrogen was proportional to the catalyst loading.

On the other hand, silica-based materials have been extensively used for anchoring biomimetic models because of their chemical robustness, stability, and versatility [233,234,235,236,237]. For example, Amaro-Gahete and group [237] synthesized a biomimetic model complex of the [FeFe]-hydrogenase active site (FeFeOH) with an ethylene bridge and a pendant hydroxyl for light-driven hydrogen production. The authors reported that the interaction of the hydroxyl group present in the complex with 3-isocyanopropyltriethoxysilane provided a carbamate triethoxysilane bearing a diiron dithiolate complex (NCOFeFe), thus becoming a potentially promising candidate for anchoring on heterogeneous supports. To prove this concept, the research group anchored into a periodic mesoporous organosilica with ethane bridges (EthanePMO@NCOFeFe) via a grafting procedure. Both molecular and heterogenized complexes were tested as catalysts for light-driven hydrogen generation in aqueous solutions. It was shown that the molecular FeFeOH diiron complex achieved a decent turnover number (TON) of 70 after 6 h, whereas NCOFeFe and EthanePMO@NCOFeFe had slightly lower activities showing TONs of 37 and 5 at 6 h, respectively. In addition, the application of mesoporous silica has been explored for anchoring biomimetic models to improve CO_2_ reduction efficiency [238]. Liang et al. [239] constructed a bioinspired artificial photosynthesis system based on ZrO_2_ nanoframes (ZFs) and metalloporphyrin to mimic the morphology of trees. The authors reported that the biomimetic system achieved an evolution yield of 35.3 μmol during a 3 h reaction with a 93.1% CO selectivity and 1.84% CO apparent quantum efficiency (*A.Q.E.*), which was about 60.8 times larger than that of pure porphyrin (Ni) (0.58 μmol). A detailed analysis revealed that the catalytic system could not only achieve fast separation of the photogenerated carriers and effective CO_2_ activation but could also possess suitable energy levels, which could efficiently transfer electrons to the Ni catalytic sites to improve its photocatalytic activity.

## 6. Conclusions

Artificial photosynthesis, drawing inspiration from nature’s ingenuity, stands at the forefront of innovative strategies for sustainable energy production and carbon management. Photoelectrochemical cells (PECs) constitute a promising avenue within this landscape. Leveraging sunlight to drive chemical reactions, PECs harbor the potential to perform both hydrogen evolution reactions (HERs) and oxygen evolution reactions (OERs), mimicking the natural photosynthetic processes. By capturing and storing solar energy as chemical energy in the form of hydrogen, PECs offer a viable route to renewable fuel production. However, as it was presented, designing efficient PECs requires a careful balance between light absorption, charge separation, and redox reaction kinetics to ensure optimum performance.

By mimicking the natural Calvin cycle, researchers are developing catalytic systems to reduce CO_2_ into usable fuels and valuable chemicals. Harnessing excess CO_2_ not only provides an alternative carbon source for chemical production but also addresses the critical issue of atmospheric CO_2_ accumulation. Although the reduction of CO_2_ is a pivotal part of artificial photosynthesis and crucial for sustainable energy and carbon management, it presents significant challenges, such as thermodynamic stability and high reduction potential, which necessitate a substantial energy input to transform it into useful compounds. The process involves multi-electron and multi-proton transfers, which, if improperly managed, can lead to a variety of products and decrease selectivity and efficiency. Moreover, the development of catalysts that can selectively direct CO_2_ towards specific products poses a major challenge as a result of the numerous potential reaction pathways. Overcoming these challenges necessitates continued research and development efforts.

In the case of biomimetic approaches, researchers have developed complexes that mimic the structure and function of the active sites in hydrogenases and photosystem II to catalyze the hydrogen evolution reaction (HER) and oxygen evolution reaction (OER), respectively. These biomimetic catalysts aim to exploit the same mechanisms utilized by their natural counterparts, thus enhancing the efficiency of these reactions. Similarly, for CO_2_ reduction, the development of biomimetic catalysts that emulate the active sites of enzymes involved in this process can provide promising solutions. This bio-inspired approach has been used to design catalysts that not only promote the conversion of CO_2_ into fuels and other useful chemicals but also improve the selectivity of the process, leading to the production of a specific desired product. Nevertheless, the design and construction of these biomimetic systems pose their own set of challenges. These include accurately replicating the complexity of natural systems, achieving a stable and efficient integration of components, and scaling up these designs for practical applications. Despite these challenges, the insights gained from studying and emulating nature’s processes hold immense potential for the future of artificial photosynthesis as it was presented.

This comprehensive review has provided an in-depth analysis of various processes associated with artificial photosynthesis, offering valuable insights into their potential applications and challenges. The exploration of light capture mechanisms, charge separation pathways, catalytic processes, and product formation has revealed significant advancements in the field while also highlighting areas that require further research. By addressing the existing limitations and focusing on material design, interface engineering, catalysis, and reactor optimization, we can pave the way for the development of efficient and sustainable artificial photosynthesis systems. Continued efforts in this direction will be instrumental in realizing the full potential of artificial photosynthesis as a promising avenue for renewable energy production.

## Figures and Tables

**Table 1:** Comparison between natural and artificial photosynthesis.

	Natural Photosynthesis	Artificial Photosynthesis	Reference
Energy Source	Sunlight	Sunlight	[1,2]
Reaction Center	Chlorophyll in photosystem	Photo-electrochemical cells	[1,2,3]
Energy Storage	Glucose (a carbohydrate)	Hydrogen or other solar fuels	[2,3,4]
Oxygen Evolution	Yes, from water	Yes, from water	[1,2,3,4,5,6]
Carbon Fixation	Yes, carbon dioxide into glucose	Potentially, carbon dioxide into carbon-based fuels	[3,4,5,6,7]
Efficiency	3–6%	Variable, still under development	[2,4]
Product Utility	Mainly food and biomass	Mainly fuels for energy and industry	[1,2,7,8]
Environmental Impact	No negative impact, reduces CO_2_	No negative impact, could reduce CO_2_	[3,5]
Catalysts	Enzymes	Man-made catalysts	[1,2,3,8]
Rate of Reaction	Relatively slow because of enzymatic constraints	Potentially faster with optimized catalysts	[1,3,9]
Operating Conditions	Ambient temperature and pressure	Variable, can be optimized for reaction	[1,2,5,7]
Evolution and Optimization	Billions of years of natural selection	Still under development, ongoing optimization	[1,2,3,4,5,6]
Dependence on Water	High, water is electron donor	High, water often used for proton/electron source	[6,7,8,10]
Lifetime/Durability	Limited by organism’s lifespan	Potentially long, dependent on material degradation	[8,9,10]

## Data Availability

The data is contained in the article and is available from the corresponding authors on reasonable request.
